# Multi-View Occluded License Plate Recognition: A Feature Fusion Method Based on Spatial Wavelet Transform

**DOI:** 10.3390/s26175414

**Published:** 2026-08-27

**Authors:** Nuo Pan, Kangshuai Zhang, Muhammad Arslan Ghaffar, Lei Peng

**Affiliations:** 1 Shenzhen Institutes of Advanced Technology, Chinese Academy of Sciences, Shenzhen 518055, China; nuo.pan@siat.ac.cn (N.P.); ks.zhang@siat.ac.cn (K.Z.); ghaffar@siat.ac.cn (M.A.G.); 2 University of Chinese Academy of Sciences, Beijing 100049, China

**Keywords:** license plate recognition, Vision Transformer, Wavelet Transformer, multi-view fusion, intelligent transportation systems

## Abstract

License plate recognition is a critical perception module in intelligent transportation systems, yet severe occlusion remains a fundamental challenge in unconstrained traffic scenes because the missing visual evidence is physically absent rather than merely degraded. Although multi-view observations provide complementary cues for recovering occluded characters, existing fusion strategies usually operate in the spatial domain and therefore suffer from feature misalignment, especially when Vision Transformers rely on local patch-wise positional encoding. To address this issue, we propose Wavelet-Enhanced Transformer, a domain-transformed multi-view recognition framework for occluded license plates. The central idea is to shift feature fusion from the spatial domain to the wavelet domain, where discrete wavelet transform decomposes encoded features into low-frequency structural components and high-frequency detail components. This transformation alleviates spatial semantic ambiguity caused by viewpoint variation and positional mismatch, enabling more robust cross-view feature aggregation. On top of this representation, we design an order-agnostic memory fusion mechanism to progressively accumulate complementary evidence from multiple partial observations, and introduce a discriminator-based stopping module to adaptively terminate unnecessary iterations for efficient deployment. Experiments on the CBLPRD-330k dataset and severe occlusion stress tests demonstrate the superiority of the proposed method. Under 20–50% character occlusion, our model maintains over 95% character accuracy and consistently outperforms mainstream baselines such as LPRNet, TrOCR, and PP-OCRv5 in character accuracy, length accuracy, and full-match rate (FMR). These results indicate that wavelet-domain feature fusion provides an effective and robust solution for highly occluded license plate recognition in complex traffic environments.

## 1. Introduction

With the continuous growth of urbanization and motor vehicle ownership, license plate recognition (LPR) has become a fundamental module for vehicle identity perception and event tracking in intelligent transportation systems, serving a wide range of scenarios such as traffic law enforcement, parking management, tolling systems, and urban security [1,2,3]. However, in real-world road environments, illumination variation, motion blur, low resolution, and viewpoint shifts all degrade recognition performance. Among these factors, occlusion is particularly challenging, because it does not correspond to a reversible degradation but rather to a genuine absence of character evidence within a single observation [2,4,5,6]. Therefore, how to stably recover complete license plate features under occlusion has become a key problem for deploying LPR in complex scenarios.

**Figure 1:**
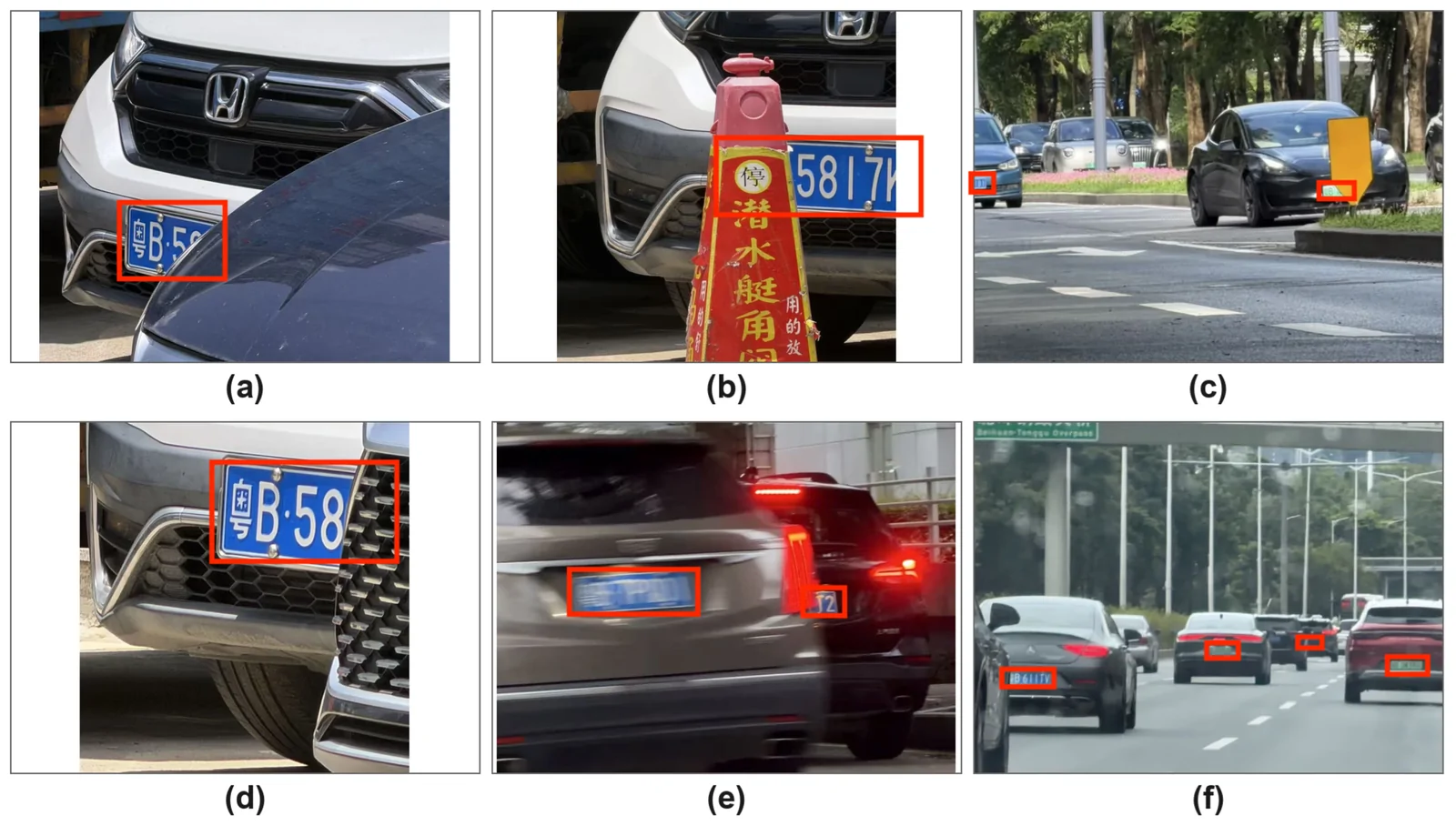
Real-world examples of partially occluded license plates (red boxes): occlusion by neighboring vehicles (**a**,**d**), a traffic cone (**b**), and a road sign (**c**), as well as degradations such as motion blur (**e**) and low resolution at highway distances (**f**). The leading character on each plate is a Chinese province abbreviation, which is an intrinsic component of the Chinese license plate number. All Chinese characters visible in the scene (e.g., on the traffic cone) are intrinsic to the real-world photographs. Such partial information loss is pervasive in traffic scenes and motivates the multi-view fusion approach of this work.

Unlike noise, blur, or compression artifacts, the core difficulty of occlusion lies in the physical irreversibility of the missing information: once a character region is occluded by a preceding vehicle, a vehicle body part, a guardrail, or an environmental object, the pixel evidence corresponding to that region effectively does not exist in the current view. For license plate recognition, this means that the model is not faced with “enhancing existing but degraded information,” but rather with “reconstructing complete features in the absence of part of the evidence.” This distinction determines that occluded license plate recognition cannot be simply equated with general image enhancement or denoising tasks. As illustrated in Figure 1, partial occlusion of license plates is ubiquitous in real traffic scenes, arising from neighboring vehicles, traffic facilities, and imaging degradations.

One intuitive approach is to leverage generative models to inpaint the missing regions. In recent years, de-occlusion, generative restoration, and diffusion models have achieved remarkable progress in a variety of vision tasks [7,8,9,10,11], providing new technical means for occluded license plate recovery. However, the essence of such methods is still to infer the missing content based on the data distribution: when the occluded area is large or the character evidence is extremely weak, the model tends to generate “plausible but actually incorrect” character results. For high-trust scenarios such as traffic law enforcement, trajectory tracking, and post hoc forensics, this prior-driven “hallucinatory recovery” carries an inherent risk. In contrast, multi-frame or multi-view observations provide more reliable, genuinely complementary evidence. When there is relative motion between the vehicle and the camera, character regions that are invisible at one moment may be re-exposed in subsequent frames or other views; different observations may also complement one another in terms of clarity, occlusion position, and local texture [12,13,14]. Therefore, a more reasonable direction for occluded license plate recognition is not to rely on a single frame to “guess” the missing characters, but to progressively recover the complete plate representation through the accumulation, selection, and fusion of multi-view fragmentary information. Based on this insight, the core problem addressed in this paper is no longer merely “whether to use multiple views,” but “how to fuse multiple views in a stable and trustworthy manner.” This problem directly determines whether multi-view information can truly be transformed into effective evidence usable for complete recognition.

Existing multi-view license plate recognition methods have already demonstrated the importance of information redundancy in mitigating occlusion. For example, character-level temporal combination, super-resolution-enhanced multi-view recognition, optical-flow-guided restoration, and feature propagation strategies all attempt to exploit cross-view complementary information to improve recognition performance [12,13,14,15,16,17]. These works show that multi-observation fusion has greater potential than single-frame recognition in handling occlusion, but they also reveal a long-overlooked problem: the vast majority of fusion still occurs in the spatial domain.

Whether through pixel-level stitching, local alignment, or directly aggregating encoded features by spatial-position correspondence, these methods implicitly assume that relevant features across different observations should be approximately aligned in spatial position. In real traffic scenes, however, this assumption is often difficult to satisfy. Variations in vehicle pose, perspective distortion, local cropping, and dynamic occlusion jointly cause the same character region to fall at completely different positions across views; conversely, different character regions may also be mapped to similar local indices because their local observation windows look alike.

This problem is particularly pronounced in Vision Transformer (ViT)-based frameworks. ViT preserves spatial relationships through patch partitioning and positional encoding, and although its self-attention modeling capability is powerful, the positional index of a patch describes the local coordinates within the current input rather than the true feature position of a character within the complete license plate [18,19,20,21]. When inputs come from different views, different cropping ranges, or different occlusion states, the feature content corresponding to the same patch index is inconsistent. If these representations are still fused directly in the spatial domain, the model tends to treat originally non-corresponding local features as alignable information and aggregate them, thereby producing cross-view feature misalignment. Therefore, the key difficulty of multi-view occluded license plate recognition is not merely “insufficient observation,” but rather “an improper choice of the representation domain in which fusion takes place.” If the fusion process still strongly depends on absolute spatial positions, then even introducing more views may weaken the utilization efficiency of complementary information due to positional misalignment. To truly unlock the value of multi-view observations, it is necessary to rethink the fusion mechanism at the representation level.

A growing body of research indicates that performing feature extraction and fusion purely in the spatial domain often struggles to simultaneously account for global semantic information and local texture details, and tends to suffer from blurred edges, loss of detail, and amplified pseudo-changes in complex scenes [22,23,24,25]. To address this, researchers have begun to introduce the wavelet transform into deep networks, explicitly modeling information at different scales and orientations by decomposing features into high-frequency and low-frequency components. Studies have shown that, when combined with deep networks, the wavelet transform exhibits good stability and information-preserving capability in tasks such as image denoising, edge preservation, deblurring, and image restoration [26,27,28,29,30,31]. These results suggest that wavelet representations can not only suppress random noise but also preserve truly discriminative local details across multiple scales. For occluded license plate recognition, this is especially important, because the model must extract trustworthy common evidence from multiple local, incomplete, and possibly misaligned observations. Based on this observation, this paper argues that multi-view occluded license plate fusion should not continue to rely on strong alignment of “position” within the spatial domain, but should instead shift toward complementary aggregation of “structure” and “detail” within the wavelet domain. In other words, this paper attempts to transform the traditionally difficult spatial alignment problem into a cross-view evidence aggregation problem within wavelet sub-bands: although different observations are inconsistent in pixel coordinates, they often still retain complementary expressions at the level of low-frequency structure and high-frequency strokes.

Accordingly, this paper proposes a Wavelet-Enhanced Transformer framework for multi-view progressive license plate recognition under occlusion. The framework first employs a ViT encoder to extract high-level semantic features of multi-view license plate images, and further maps the features from the spatial domain to the wavelet domain, achieving cross-view information fusion at the sub-band level; subsequently, an order-agnostic memory accumulation mechanism is used to progressively integrate the fragmentary evidence from different observations. Finally, a discriminator module is introduced to adaptively determine whether the current information is sufficient to support complete recognition, thereby reducing unnecessary iterative computation overhead. Unlike traditional methods that focus only on the final recognition result, our method simultaneously models the two key questions of “how information progressively becomes sufficient” and “when the system can stop observing.” From a methodological perspective, the core innovation of this paper is neither simply increasing the number of multi-view inputs nor making local improvements to existing spatial-domain fusion strategies, but rather proposing a multi-view fusion paradigm that migrates from the spatial domain to the wavelet domain. This paradigm provides a new modeling framework for mitigating cross-view feature misalignment under occlusion, and also offers a methodological basis for analyzing the performance boundary under extreme occlusion. The main contributions of this paper are summarized as follows:

1. We design a progressive recognition method that combines ViT encoding, wavelet-domain fusion, and order-agnostic memory accumulation, enabling the continuous integration of fragmentary evidence from different observations.
2. We propose a wavelet-domain multi-view fusion framework for occluded license plate recognition, which reduces the dependence of cross-view fusion on explicit spatial alignment through decoupled modeling of sub-band structure and detail information.
3. We conduct systematic experiments under different occlusion intensities and against multiple categories of baseline models, validating the advantages of the proposed method in recognition performance and further analyzing the relationship between multi-view evidence accumulation and the recognition boundary.

The remainder of this paper is organized as follows: Section 2 reviews related work on occluded license plate recognition, multi-view fusion, Transformer modeling, and transform-domain representations; Section 3 presents the proposed spatial-to-wavelet-domain progressive fusion framework and its key modules; Section 4 provides the experimental setup, main experiments, boundary experiments, and interpretability analysis; and Section 5 concludes the paper and discusses future work.

## 2. Related Work

With the rapid development of intelligent transportation systems (ITS), license plate recognition technology has been widely applied in scenarios such as vehicle management, traffic law enforcement, and parking tolling [3]. However, in real-world complex traffic environments, license plate occlusion remains one of the key factors affecting recognition performance. To address this problem, existing studies have mainly proceeded along the directions of single-frame image restoration, multi-view information fusion, Transformer-based modeling, and signal-processing-domain transformation.

Early methods mostly focused on single-frame occlusion restoration. Liu [32] proposed a progressive vehicle re-identification framework for the vehicle re-identification task and incorporated a license plate recognition module into the system; building on this, combining single-stage character segmentation with adversarial super-resolution methods can significantly improve the legibility of low-quality license plate images [33]. Yueh-Tse [34] further compared the performance of several adversarial networks in license plate image reconstruction, providing new insights for the restoration of low-quality and occluded license plates. Such methods are effective to some extent under mild occlusion or image degradation, but they essentially still rely on generative models to infer the missing content, and tend to produce character misrecognition or incorrect digit-count estimation under severe occlusion.

Consequently, research has gradually shifted toward exploiting multi-frame or multi-view information for fusion-based recognition. Unlike single-view restoration, multi-view methods leverage the observational redundancy formed during vehicle motion to extract complementary information from different viewpoints, thereby mitigating the information loss caused by occlusion. The multi-view radar–camera fusion method MVFusion, proposed by Z. Wu [35], enhances the correlation among heterogeneous sensors through feature alignment and a cross-attention mechanism, achieving good results in 3D object detection. Its core idea demonstrates that the attention mechanism can dynamically allocate information weights across multi-view inputs, thereby highlighting key observations and suppressing redundant interference. This idea is equally applicable to occluded license plate recognition: when the license plate regions corresponding to different views exhibit differences in visibility, a well-designed cross-view fusion strategy helps recover the complete features. Nevertheless, existing license plate recognition systems still primarily rely on positional relationships in image space for modeling—for example, character segmentation depends on the relative arrangement of characters, and OCR recognition depends on complete or mostly complete character contours. When a license plate is heavily occluded, such critical spatial information is prone to loss or misalignment, leading to a significant drop in system accuracy. To this end, transforming the feature representation from the spatial domain into a transform domain more suitable for fusion provides a new way to alleviate the spatial-dependence problem.

### 2.1. Vision Transformer

In 2020, Google Research proposed the Vision Transformer [20], which for the first time directly applied a pure Transformer architecture to image recognition tasks, driving a paradigm shift in computer vision from convolution-based modeling toward global self-attention modeling. Subsequently, the Swin Transformer [36] effectively reduced the computational overhead of the standard ViT on high-resolution images through a hierarchical windowed attention mechanism, becoming one of the mainstream backbone networks for object detection and image segmentation tasks.

Although ViT exhibits powerful global modeling capability in visual recognition tasks, its limitations are equally evident [36,37,38]. First, the computational complexity of the self-attention mechanism is $O(N^{2})$, resulting in high training and inference costs under high-resolution inputs. Second, ViT relies heavily on large-scale data pre-training and is generally less stable than CNNs in few-shot scenarios. Third, ViT lacks the local inductive bias and translation invariance that CNNs naturally possess, and thus often requires additional design to compensate for these weaknesses in small-size images or local fine-grained tasks.

Despite this, the Transformer remains highly suitable for license plate recognition tasks. On the one hand, its global dependency modeling capability helps handle the contextual relationships among characters and irregular layouts; on the other hand, its attention-based structure also facilitates information fusion in multi-view and multi-modal scenarios. Therefore, ViT and its variants have been widely used in tasks such as license plate detection, character recognition, and multi-sensor fusion, and exhibit good robustness under occlusion. Meanwhile, to address the issue of high computational cost, optimization methods such as local windowed attention have made ViT more deployable in practical systems.

### 2.2. Positional Encoding Issues in Spatial-Domain Multi-View ViT

In the Transformer architecture, the self-attention mechanism is inherently permutation-invariant; without explicitly introducing positional information, the model cannot distinguish the ordering relationships among input elements. Therefore, existing studies typically employ absolute positional encoding or learnable positional encoding to supplement spatial information, such as the absolute positional encoding proposed in “Attention Is All You Need” [21], as well as the subsequently widely used learnable positional embedding:

(1) $${\mathrm{PE}}_{\mathrm{learn}}\left({\mathrm{Plate}}_{\mathrm{pos}},i\right)=E_{\mathrm{pos},i},$$

Here, pos denotes the positional index (e.g., partitioning the license plate image into $H_{\mathrm{patch}}\times W_{\mathrm{patch}}$ image patches), *i* is the embedding-dimension index, and *E* is a learnable parameter matrix used to learn a corresponding vector representation for each position pos. However, in the multi-view occluded license plate recognition task, ViT positional encoding suffers from a key drawback: the positional encoding of a standard ViT is based on the local index of a patch within the current input image, rather than the true physical position of a character within the complete license plate. When the input is a locally cropped image from a partial view, different physical regions may be mapped to the same local index position, thereby causing spatial feature confusion during feature fusion. Specifically, suppose there are *N* input images from *N* views, $V_{1},V_{2},V_{3},\ldots ,V_{N}$, where each $V_{n}$ is a local observation of $I_{\mathrm{global}}$ and contains only partial license plate information. In the standard ViT, image $V_{n}$ is partitioned into *K* patches, and we denote the *i*-th patch of the *n*-th view as $x_{i}^{n}$. The input embedding of the ViT is:

(2) $$z_{i}^{n}=\mathrm{Embed}\left(x_{i}^{n}\right)+{\mathrm{PE}}_{\mathrm{learn}}^{i},$$

Here, $z_{i}^{n}$ is the final input vector of the *i*-th patch in the *n*-th view, Embed(·) is the linear projection layer, and ${\mathrm{PE}}_{\mathrm{learn}}^{i}$ is the locally approximate positional encoding. Formally, let $\Phi_{n}\left(i\right)$ denote the mapping from the local patch index *i* in view *n* to the global license plate coordinate system. For two views $V_{a}$ and $V_{b}$ that capture different parts of the license plate (i.e., $\Phi_{a}\left(i\right)\neq \Phi_{b}\left(i\right)$), the ViT encoder erroneously assigns them the same positional embedding: ${\mathrm{PE}}_{\mathrm{learn}}^{i}\left(a\right)={\mathrm{PE}}_{\mathrm{learn}}^{i}\left(b\right)$. This introduces a form of spatial feature confusion, in which visual features from spatially distinct regions (e.g., the first character and the last character) are mapped to the same positional representation in the latent space, thereby hindering the effective stitching of the complete license plate sequence.

### 2.3. Wavelet Transform

Signal processing techniques provide a perspective on visual information representation and fusion that differs from traditional spatial-domain modeling. Common transformation methods include the wavelet transform. For occluded license plate recognition, the significance of such methods lies in the fact that, by mapping features from the spatial domain to the frequency or time–frequency domain, they can alleviate the spatial-position dependence to a certain extent and provide a new representational basis for multi-view feature alignment.

The wavelet transform (WT) combines localized analysis capability with multi-scale representation capability: it inherits the localization concept of the short-time Fourier transform while overcoming the limitations imposed by a fixed window, and is therefore particularly suitable for visual tasks involving local occlusion, missing details, and non-stationary characteristics. In recent years, the combination of the wavelet transform with neural networks has become a research hotspot: one class of methods uses the wavelet transform as a preprocessing means for feature extraction, while another class deeply embeds the wavelet transform into the network architecture, forming a jointly optimized framework [39,40].

The wavelet transform has achieved good results in tasks such as image denoising, edge detection, deblurring, image restoration, and compression [26,27,28,29,30,31,41]. For example, in image denoising tasks, the wavelet transform helps suppress noise while preserving more details [26,27]; in deblurring tasks, it can effectively assist the model in recovering high-frequency texture information and reduce training complexity [28,29,30,31]; and in image compression and high-resolution activation compression tasks, the wavelet representation also exhibits good information-preserving capability [41]. These studies show that the wavelet transform can not only effectively decompose the low-frequency structure and high-frequency details of an image, but also possesses the potential to work synergistically with deep networks, thereby providing a promising technical pathway for multi-view occluded license plate feature fusion.

Overall, multi-view feature fusion has become an important direction for handling environmental variation, viewpoint variation, and local occlusion, while transform-domain representations in signal processing—especially wavelet-domain representations—offer a new solution for alleviating feature misalignment and semantic confusion in the spatial domain.

## 3. Method

The proposed Wavelet-Transform-Enhanced Fusion (WTEF) framework targets the multi-view occluded license plate recognition task. Its core objective is to aggregate complementary features from different views in the wavelet domain and to perform license plate character recognition in the reconstructed visual feature space. This section first introduces the overall system framework, describing the relationships among the multi-view input, the ViT encoder, the wavelet-domain fusion module, the inverse wavelet reconstruction, and the character decoder; it then presents the spatial-to-wavelet-domain transformation mechanism and analyzes the roles of low-frequency structural information and high-frequency detail information in occluded license plate recognition; and finally, it introduces the WTEF fusion module based on a multi-view set input, explaining its cross-view complementarity, permutation invariance, and progressive feature completion mechanism.

### 3.1. Overall System Framework

**Figure 2:**
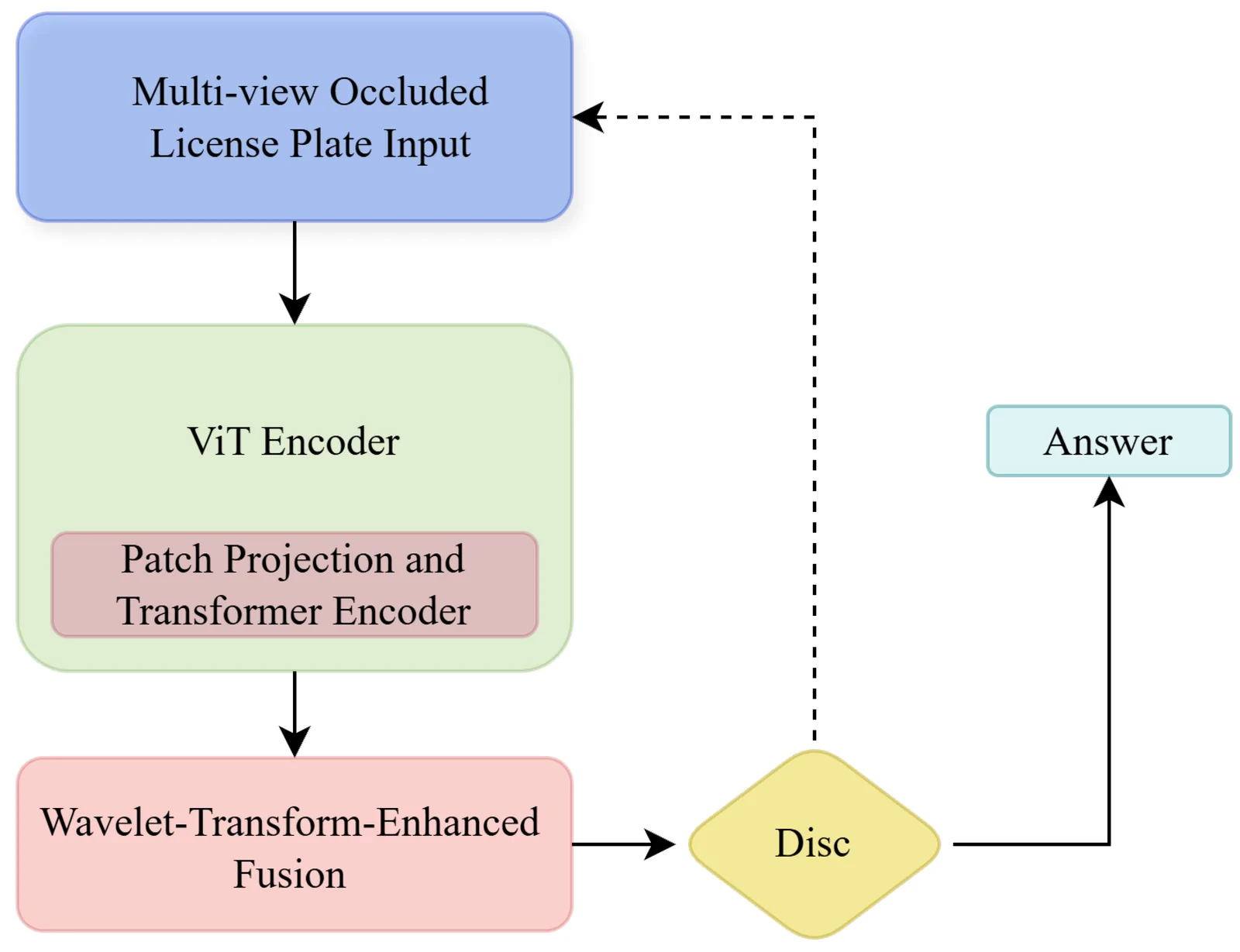
Overall architecture of the proposed WTEF framework. Multi-view occluded plate images are independently encoded by a ViT encoder, decomposed via DWT into sub-bands, fused in the wavelet domain, reconstructed via IDWT, and finally fed to the character decoder and the Discriminator (Disc) for progressive recognition.

The WTEF framework formulates occluded license plate recognition as a multi-view feature aggregation task. The overall data flow is shown in Figure 2: first, the multi-view input set of the same license plate is passed through a ViT encoder to extract visual features; subsequently, the features of each view are decomposed by the wavelet transform into low-frequency structural and high-frequency detail sub-bands, and cross-view fusion is completed within the WTEF module. Finally, the model output is fed into a discriminator, Disc, which determines whether the multi-view information is sufficient to recover the complete license plate. If not, the system continues to receive new view information and updates the fused features. In this way, WTEF forms a closed-loop data flow of “multi-view input, ViT encoding, DWT decomposition, wavelet-domain fusion, and character recognition.”

### 3.2. Patch Projection and Transformer Encoding

**Figure 3:**
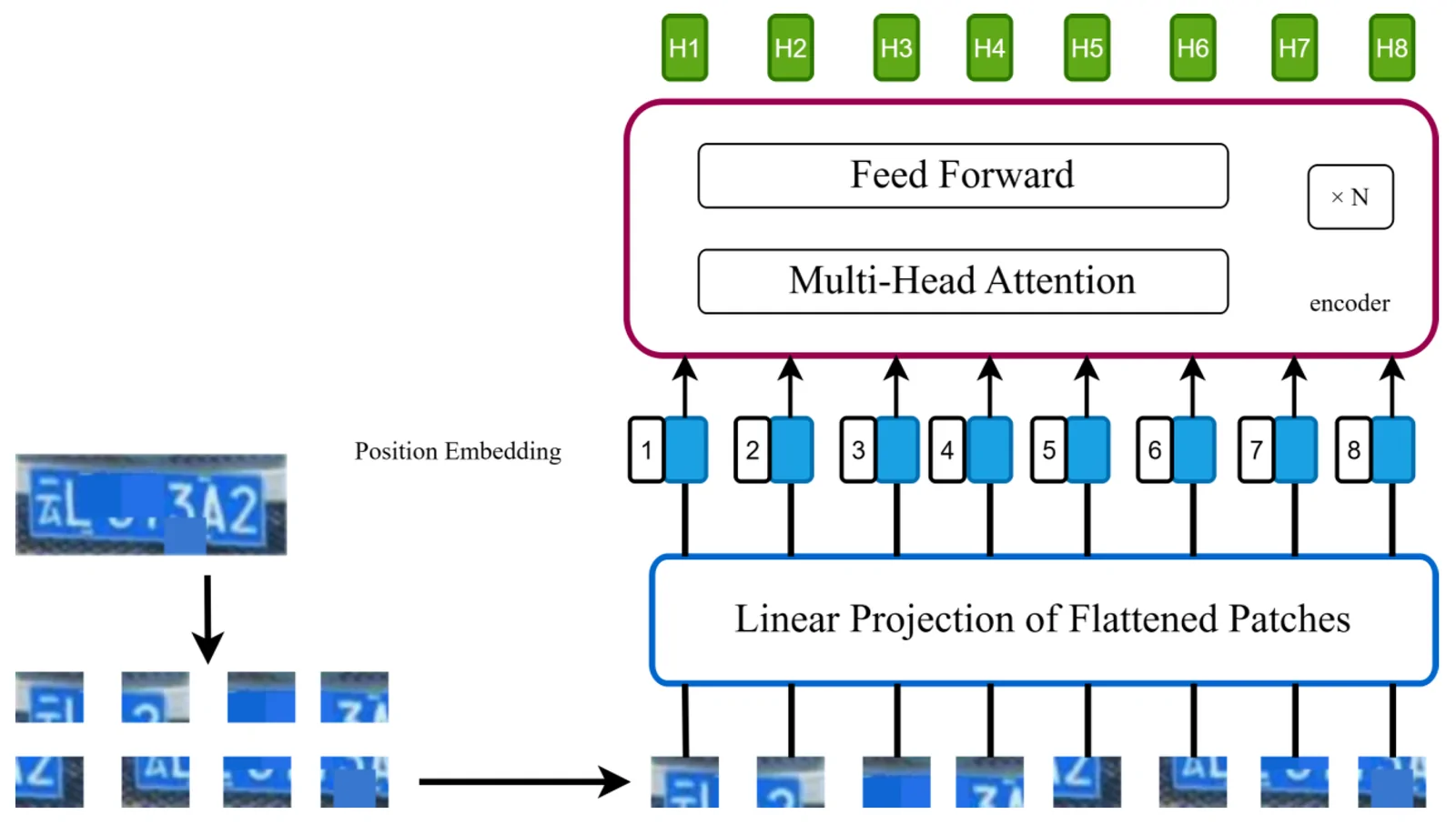
Patch embedding and Transformer encoding. The input image (H×W) is divided into L=(H/p)×(W/p) non-overlapping p×p patches, each flattened and linearly projected to a *D*-dimensional token; a learnable positional encoding is added before the Transformer encoder. The leading character on the sample plate is a Chinese province abbreviation, an intrinsic part of the license plate number.

Let the currently available multi-view input set be:

(3) $$I={\left\{I^{\left(v\right)}\right\}}_{v=1}^{V},\;I^{\left(v\right)}\in R^{3\times H\times W},$$

where *v* denotes the view index and *V* denotes the number of currently acquired views. Each view image is uniformly resized and fed into the encoder. The encoder first partitions the input image into L=(H/p)×(W/p) non-overlapping p×p patches (in this work, p=8), flattens each patch, and maps it to a *D*-dimensional vector through a shared linear projection layer, after which a learnable positional encoding *P* is added (Figure 3):

(4) $$Z_{0}^{v}=\mathrm{flatten}\left(X^{\left(v\right)}\right)\cdot W_{\mathrm{proj}}+b_{\mathrm{proj}}+P\in R^{L\times D}.$$

Subsequently, *N* layers of Multi-Head Self-Attention and Feed-Forward modules iteratively model the global dependencies among license plate characters, outputting the high-dimensional visual features corresponding to each view:

(5) $$F^{\left(v\right)}=E_{\theta }\left(I^{\left(v\right)}\right)\in R^{L\times D}.$$

### 3.3. Transformation from the Spatial Domain to the Wavelet Domain

In license plate recognition tasks, license plate characters typically exhibit elongated and locally pronounced structures, whereas occlusion, variations in shooting angle, and detection-box offsets cause the character strokes to be difficult to strictly align with the fixed patch grid in the spatial domain. In particular, during patch-based feature encoding, a single character stroke may be split across multiple adjacent patches, or multiple local structures may be compressed into the same patch, thereby weakening the model’s ability to represent fine-grained information such as character edges, stroke breaks, and occlusion boundaries.

A two-dimensional discrete wavelet transform (DWT) could in principle be applied to the input image before encoding, so that the encoder operates on frequency-decomposed sub-bands—a strategy with a long history in wavelet–network integration [39,40]. In our setting, however, this ordering is suboptimal. The cross-view positional misalignment discussed in Section 2.2 is most severe at the pixel level, where the same character pixel falls at very different coordinates across views; a DWT applied to the raw image would therefore decompose already misaligned pixel content rather than the underlying character structure. The ViT encoder maps the input into a semantic token space in which this misalignment is substantially reduced, and only at this level does a DWT separate semantic structure (the plate-region consensus across views) from semantic detail (character edges, stroke boundaries, and occlusion breaks), rather than pixel-level intensity from pixel-level texture that the encoder would otherwise have to re-encode. Operating after encoding is also computationally negligible: the encoder reduces the input to a compact feature tensor on which a Haar DWT is trivial, whereas a DWT on the raw image would yield a four-channel tensor that still has to pass through the encoder. We therefore apply the DWT to the encoded features of each view—encoding first, then screening by frequency sub-band, consistent with prior feature-level wavelet integration [42,43]—and decompose them into a low-frequency structural sub-band and three directional high-frequency detail sub-bands:

(6) $$C^{\left(v\right)}=\mathrm{DWT}\left(F^{\left(v\right)}\right)=\left\{LL_{v},LH_{v},HL_{v},HH_{v}\right\},$$

where $LL_{v}$ encodes the main structure and global semantics of the license plate, and $LH_{v}$, $HL_{v}$, and $HH_{v}$ respectively preserve the detail responses of character edges, stroke boundaries, and occlusion breaks in the horizontal, vertical, and diagonal directions.

We adopt the wavelet transform, whose core aim is to perform multi-scale refinement analysis of a function or signal through scaling and translation operations. To better process the feature vectors after Transformer encoding, we select the discrete wavelet transform: after the encoder operation $Z_{\mathrm{out}}=\mathrm{ViT}\left(X_{\mathrm{in}}\right)$, the resulting feature tensor $Z\in R^{H\times W\times C}$ contains high-level abstract semantics of the image. To facilitate the effective fusion of multi-view features, we apply a two-dimensional discrete wavelet transform to it. Specifically:

(7) $$\begin{array}{cccc} X_{LL} & =\left({\left(X*h\right)}_{\mathrm{row}}\;{↓}_{2}\right)*h_{\mathrm{col}}{\;↓}_{2}, & X_{LH} & =\left({\left(X*h\right)}_{\mathrm{row}}\;{↓}_{2}\right)*g_{\mathrm{col}}{\;↓}_{2},\\ X_{HL} & =\left({\left(X*g\right)}_{\mathrm{row}}\;{↓}_{2}\right)*h_{\mathrm{col}}{\;↓}_{2}, & X_{HH} & =\left({\left(X*g\right)}_{\mathrm{row}}\;{↓}_{2}\right)*g_{\mathrm{col}}{\;↓}_{2}, \end{array}$$

where $X_{LL}$ is the low-frequency approximation component, and $X_{LH}$, $X_{HL}$, and $X_{HH}$ are the high-frequency detail components in the horizontal, vertical, and diagonal directions, respectively; the spatial resolution of each sub-band is $\frac{H}{2}\times \frac{W}{2}$. In this work, the Haar wavelet is adopted as the basis function, with filters $h=\frac{1}{\sqrt{2}}\left[1,1\right]$ and $g=\frac{1}{\sqrt{2}}\left[1,-1\right]$.

When the wavelet transform is applied to the feature maps of the encoder, the meaning of “frequency” is lifted to a higher level on top of the feature maps, reflecting the “spatial rate of change of the corresponding features.” In the feature space, the low-frequency component ($X_{LL}$) corresponds to regions where the values vary smoothly across the feature map; for the license plate recognition scenario, it encodes the main structure of the license plate and the background context (e.g., the approximate region of the plate in the feature space), reflecting the global feature consensus captured across different views. In contrast, the high-frequency components ($X_{LH}$, $X_{HL}$, $X_{HH}$) correspond to regions where the values change drastically across the feature map, representing feature edges and semantic transition boundaries. Through wavelet decomposition, the image features are divided into sub-bands of different frequencies, facilitating structured representation in the vector space model.

For the low-frequency component, due to the misalignment of ViT positional encoding, the features from different views exhibit slight spatial offsets, yet the semantic information is redundant and stable. Average fusion can suppress noise and extract the multi-view “semantic consensus” as the baseline for feature alignment. Similarly, the high-frequency components contain semantic detail information; under occlusion, the missing details can be supplemented using the approximate semantic information from different views. Related studies have shown [44,45] that frequency-domain transformation helps extract blurred semantic information in images. Therefore, this paper proposes to decompose different semantic features into their corresponding frequency intervals by designing a reasonable mapping function. After completing the transformation from the “visual” domain to the “wavelet” domain, the different frequency components can be effectively separated, thereby supporting semantic-level fusion in the frequency domain. In summary, the features extracted by ViT form a cluttered and misaligned jigsaw puzzle; the low-frequency information extracts the blurred outline of the puzzle, while the high-frequency information extracts its contour lines. Through this separation strategy, the problem of poor stitching performance under direct positional misalignment can be resolved.

### 3.4. Cross-View Fusion in the Wavelet Domain

**Figure 4:**
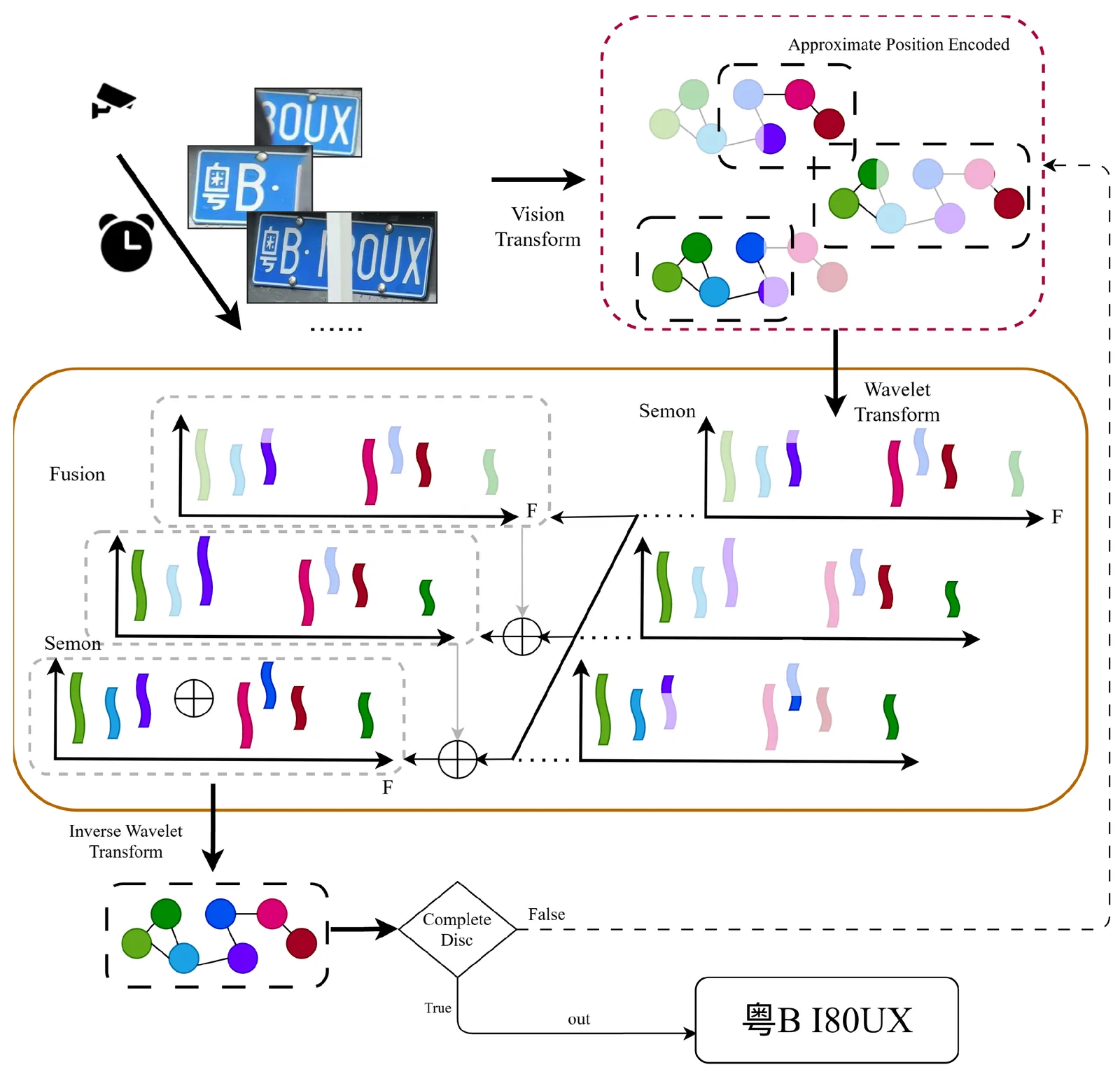
Occluded license plate multi-view fusion framework. Colored wave segments denote feature fragments from different views; colors are used only to distinguish views and carry no quantitative meaning. The leading character on each plate is a Chinese province abbreviation, an intrinsic component of the license plate number.

In the multi-view occluded license plate recognition scenario, different views often contain complementary information. On the one hand, a character region occluded in one view may possess a more complete visible structure in other views; on the other hand, there are also differences in illumination, blur level, and local distortion across views. If the multi-view features are fused directly in the spatial domain, the model is prone to being affected by local positional offsets and patch alignment errors between views, making it difficult to effectively aggregate the complementary information. The overall pipeline of the proposed multi-view fusion framework is illustrated in Figure 4.

The input feature vectors pass through convolution operations, where the spatial size is preserved and the number of channels increases. It should be noted that, in the Feature Enhancement (FE) module, all convolution kernels have a size of 5×5 except for the last one, which has a size of 1×1. The FE module follows the convolutional design paradigm of YOLO [46], where stacked large-kernel convolutions enlarge the receptive field to capture character-level local structures while preserving spatial resolution, and a final 1×1 convolution performs channel-wise feature integration.

For multiple occluded license plate inputs, the input signal is a linear combination of multiple signals, $f\left(t\right)=\alpha_{1}f_{1}\left(t\right)+\alpha_{2}f_{2}\left(t\right)+\cdots +\alpha_{n}f_{n}\left(t\right)$, and the wavelet transform satisfies:

(8) $$Wf\left(t\right)=\alpha_{1}Wf_{1}\left(t\right)+\alpha_{2}Wf_{2}\left(t\right)+\cdots +\alpha_{n}Wf_{n}\left(t\right).$$

This directly conforms to the definition of a linear transformation, $T\left(\alpha f_{1}+\beta f_{2}\right)=\alpha T\left(f_{1}\right)+\beta T\left(f_{2}\right)$. Since the DWT is a linear operator, the weighted summation of multi-view wavelet coefficients is equivalent to “first weighting the original multi-view images and then performing wavelet decomposition,” thereby ensuring that the fusion possesses an interpretable equivalence relationship between the wavelet domain and the spatial domain.

For license plate information appearing at any count or time instant, we fuse all of it. Owing to the commutativity and associativity of addition, regardless of the order in which the multi-view observations enter the fusion module, the finally accumulated wavelet-domain memory remains the same; the model is therefore permutation-invariant with respect to the input sequence, reflecting temporal order-agnosticism. Accordingly, in our design, we adopt a spatiotemporally decoupled fusion approach. Unlike RNNs with nonlinear gating, this paper employs a memory mechanism in the form of linear accumulation: in the wavelet domain, feature superposition maintains linear separability, avoiding the view bias introduced by nonlinear gating; meanwhile, owing to the commutativity of addition, the fusion process is naturally permutation-invariant with respect to the frame sequence:

(9) $$h_{t}=\frac{t-1}{t}h_{t-1}+\frac{1}{t}f_{t},$$

where $h_{t-1}$ represents the historical memory and $f_{t}$ is the current observation. This design is structurally similar to a recurrent network: by continuously ingesting fragmentary license plate features $f_{t}$, it progressively completes the global representation $h_{t}$. This “recurrent-like” structure exhibits significant robustness: it is not only insensitive to the order of input frames but also decouples the spatial position of occlusion at the feature-dimension level. This means that the model can automatically complete the stitching process from local fragments to complete license plate features through feature superposition in the latent space.

**Figure 5:**
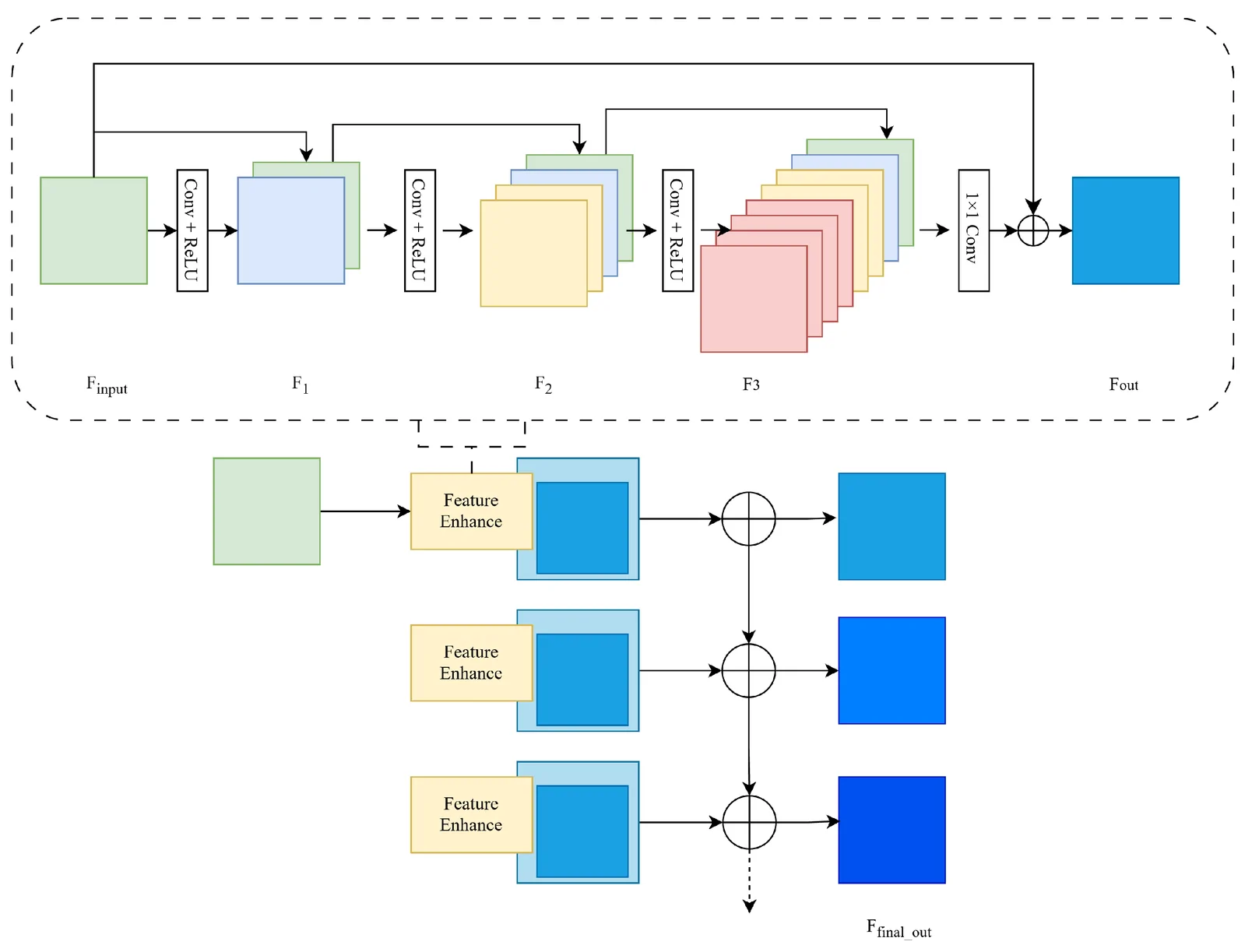
Architecture of the proposed Wavelet-Transform-Enhanced Fusion (WTEF) module. including a zoomed-in view of the internal structure of the Feature Enhancement (FE) submodule, and the overall architecture of the WTEF module, in which per-view features are enhanced by FE and progressively accumulated through linear fusion.

The overall process of this module, illustrated in Figure 5, can be described as follows:

(10) $$\mathrm{WTEF}\left(x\right)=f_{\mathrm{IDWT}}\left(F_{\mathrm{fusion}}\left(f_{\mathrm{FE}}\left(f_{\mathrm{DWT}}\left(f_{\mathrm{ViT}}\left(x\right)\right)\right)\right)\right),$$

where WTEF(·) denotes the end-to-end mapping of the proposed Wavelet-Transform-Enhanced Fusion module, i.e., the composition of the following sub-functions: $f_{\mathrm{ViT}}$, which denotes the ViT-based encoder function, $f_{\mathrm{DWT}}$ and $f_{\mathrm{IDWT}}$, which denote the DWT (wavelet transform) and IDWT (inverse wavelet transform) functions, $F_{\mathrm{fusion}}$, which denotes the fusion function, and $f_{\mathrm{FE}}$, which denotes the FE module.

**Figure 6:**
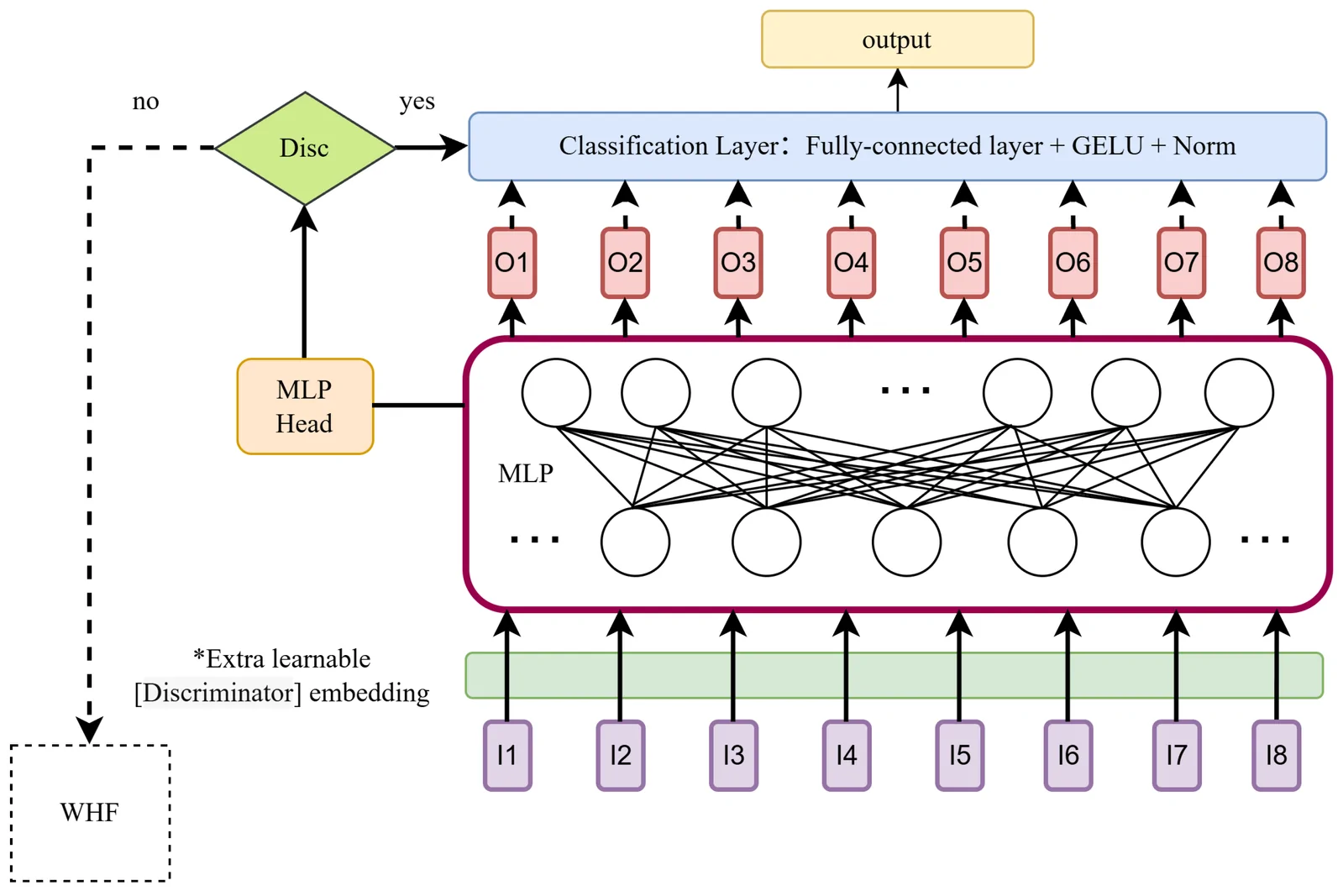
Discriminator-guided iterative feature enhancement and recognition. The input token sequence $I_{1},\ldots ,I_{n}$ denotes the fused feature tokens produced by the WTEF module after inverse wavelet reconstruction (n=96 for a 48×128 input with 8×8 patches), rather than the raw occluded images; raw views are consumed only at the patch-embedding stage (Figure 3). The asterisk (*) marks the extra learnable discriminator embedding appended to the fused token sequence.

Finally, the fused wavelet-domain features are remapped back to the visual space through the inverse wavelet transform to reconstruct the complete license plate image. The inverse wavelet transform is essentially a convolution process between the wavelet basis functions and the obtained coefficients, and is a key step for ensuring the recoverability of wavelet-domain processing results; it has been widely applied in fields such as signal processing, image compression, and lossless sampling [47]. By continuously superimposing the fused features and performing the inverse transform, the model can effectively complete the reconstruction of the image features, thereby recovering the complete license plate information. The reconstructed feature tensor is then unfolded into the token sequence $I_{1},\ldots ,I_{n}$ in Figure 6; i.e., the output of WTEF(x) in Equation (10) serves as the input to the character decoder and the discriminator.

### 3.5. Inverse Wavelet Reconstruction and Character Decoding

The fused wavelet coefficients are remapped back to the visual feature space through the inverse wavelet transform:

(11) $$F=\mathrm{IDWT}(C_{f}).$$

The reconstructed feature *F* is fed into the decoding layer, and the classification head outputs the license plate character sequence:

(12) y=ClsHead(F),

where ClsHead consists of fully connected layers, a GELU activation, and mean pooling. In parallel, the fused feature is also fed into the integrity discriminator Disc to determine whether the currently accumulated views are sufficient to recover the complete license plate.

The fused feature tensor output by the WTEF module is remapped from the “wavelet domain” back to the “ViT visual feature domain” through the inverse wavelet transform, and already contains the complete license plate semantic information after cross-view completion. Therefore, the decoding stage does not require an additional autoregressive Transformer decoder; a single feed-forward mapping of *F* is sufficient to recover the complete license plate characters. Specifically, this paper adopts a lightweight classification head ClsHead composed of fully connected layers, GELU activation, and mean pooling, which independently outputs the category distribution for each character position. This design is based on two priors of license plate characters: (i) there is no contextual dependency among characters in the natural-language sense, as they are mostly random combinations of letters and digits; and (ii) the character length is relatively fixed. Therefore, compared with the masked autoregressive decoding approach in conventional OCR, the parallel classification head significantly reduces the inference overhead while maintaining recognition accuracy.

### 3.6. Integrity Discriminator Disc

Analogous to the [CLS] token in ViT, we append an extra learnable discriminator embedding to the fused token sequence; it aggregates sequence-level evidence, and its output representation is read by the MLP head to drive the completeness judgment of the discriminator. The discriminator takes the output of an additional vector head, MLPHead, as input, and is used to determine whether the currently fused multi-view features are sufficient to reconstruct the complete license plate. The discrimination process is defined as:

(13) $$z=\mathrm{MLPHead}\left(F\right),\;p=\sigma \left(z\right)=\frac{1}{1+e^{-z}}.$$

(14) Disc(F)=1[p≥τ]∈{no,yes},

where z∈R is the scalar output of the final linear layer of the MLPHead, p∈[0,1] denotes the probability that the currently accumulated views are sufficient to recover the complete license plate, and τ is the discrimination threshold. When Disc(F)=yes, the system outputs the character predictions from the classification head and terminates inference; otherwise, it waits for a new view observation to enter the WTEF module, updates the cross-view fusion memory, and performs discrimination again.

As shown in Figure 6, given the input token sequence, the model additionally introduces a learnable discriminator embedding and feeds it, together with the character tokens, into the MLP encoder. The character tokens output by the MLP are sent to the classification layer to predict the category probabilities for each character position; meanwhile, the discriminator token passes through the MLPHead to generate a reliability score for the current feature representation. When the discrimination confidence exceeds the preset threshold, the model directly outputs the recognition result; otherwise, the current feature is fed back for further enhancement, and the recognition process is re-executed until a trustworthy prediction is obtained or the maximum number of iterations is reached.

## 4. Experiment Settings and Result Analysis

### 4.1. Experimental Setup

#### 4.1.1. Data Preparation

**Figure 7:**
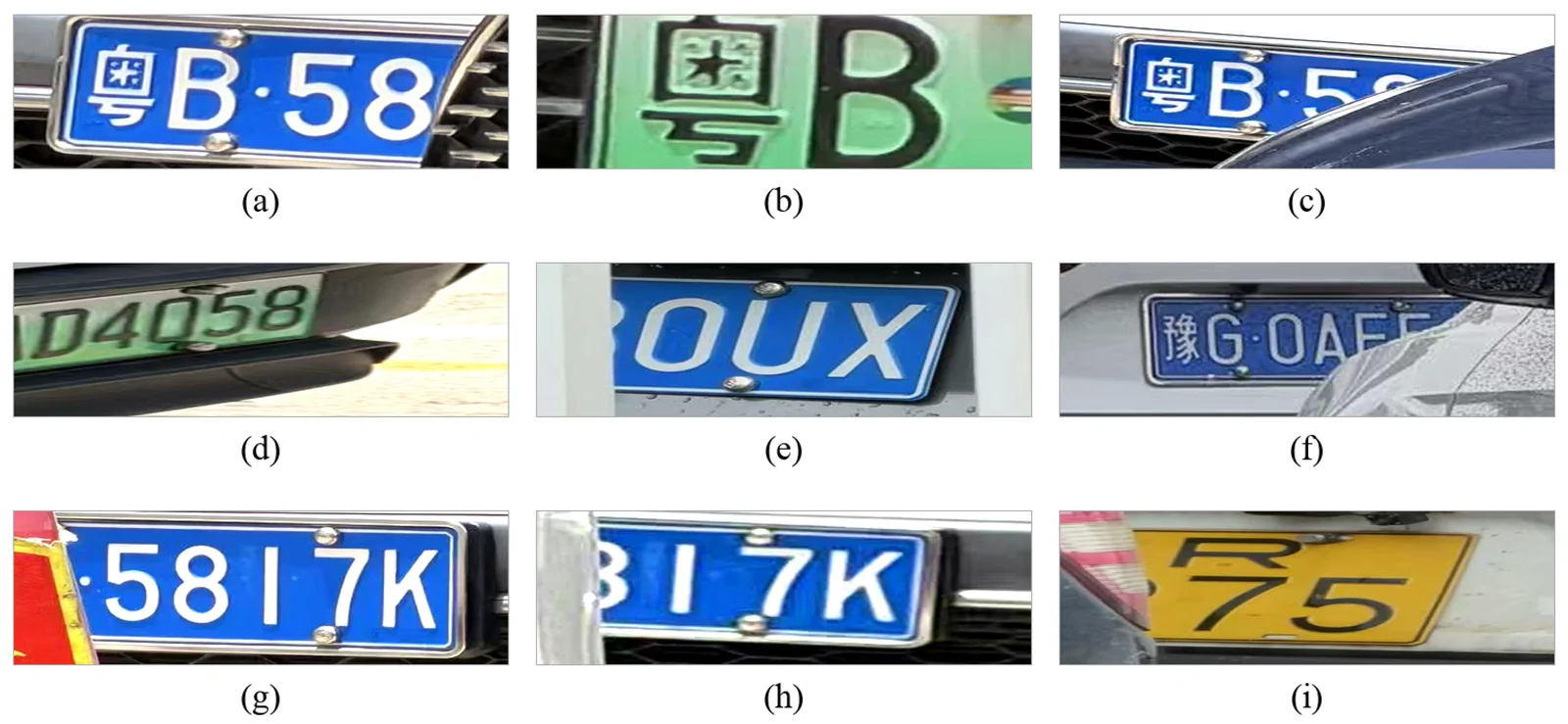
Samples from the real-world occluded license plate dataset. The nine samples cover diverse real occluders and plate types, reflecting the illumination, blur, and distortion variations encountered in real multi-view capture: (**a**,**c**,**f**,**h**) blue or new-energy green plates partially blocked by adjacent or foreground vehicles; (**b**,**d**) new-energy green plates partially out of the field of view; (**e**) a blue plate behind barrier rails; (**g**) a blue plate occluded by a traffic cone; (**i**) a yellow plate on a truck body. The leading character on each plate is a Chinese province abbreviation, which is an intrinsic component of the Chinese license plate number.

The training set used in this study is an augmented dataset composed of two complementary parts. The first part is the CBLPRD-330k [48] dataset, which serves as the source domain for training. This dataset provides a robust foundation of 330,000 synthetic images generated through GANs, ensuring high fidelity and diverse visual conditions. Unlike datasets collected from surveillance cameras which may suffer from uneven class distribution, CBLPRD-330k offers a balanced distribution across all character classes. Although the generation process results in some license plate numbers that do not conform to real-world issuance regulations, this character-level independence is advantageous for training robust character recognition backbones, ensuring equal attention to every potential character class regardless of its frequency in real-world scenarios. The second part consists of real occluded multi-view license plate images captured at urban traffic intersections, which are also included in training, so that the differences in illumination, blur level, and local distortion encountered in real multi-view capture are directly carried into the training distribution by these real samples, as illustrated in Figure 7.

To verify the robustness of the proposed method under complex occlusion conditions, this paper further constructs a controllable occlusion experimental subset based on the CBLPRD-330k dataset. Since the license plate images in the original dataset are all occlusion-free samples, this paper uniformly introduces a random occlusion injection mechanism during both the training and testing stages to generate occluded observations of varying intensities. Specifically, for each license plate image, it is first partitioned into a regular grid of 20 columns horizontally and 5 rows vertically, yielding a total of 100 grid cells; a subset of grids is then randomly selected for occlusion according to a preset occlusion rate, thereby forming input samples with local missing regions, cross-region occlusion, and non-uniform visibility.

**Figure 8:**
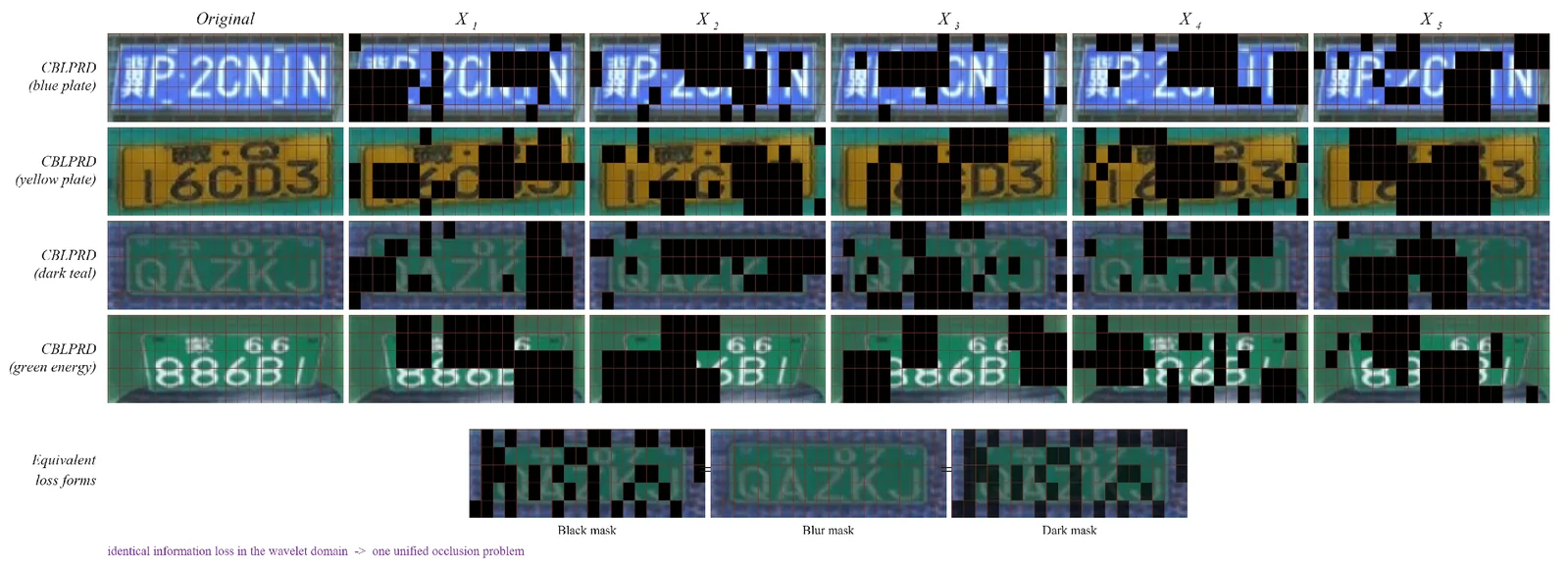
Occlusion generation results on representative CBLPRD-330k training samples (40% occlusion). The four rows show different plate types (blue, yellow, dark-teal, and new-energy green plates), illustrating the diversity of plate type, illumination, rotation, and rendering conditions inherent to the dataset; columns show the original plate and five views $X_{1}$–$X_{5}$ with independent random occlusion masks. Bottom: identical regions masked by black blocks, blur, or darkness constitute equivalent information loss in the wavelet domain. The leading character on each plate is a Chinese province abbreviation, which is an intrinsic component of the Chinese license plate number.

The reason for adopting this controllable injection mechanism is that the occlusion ratio of real occluded plates is difficult to control and measure precisely, whereas the CBLPRD-330k subset allows the effect of the occlusion rate (20–50%) to be quantified under controlled conditions. The two data sources thus complement each other, with real samples providing realistic degradations and the synthetic subset providing quantifiable occlusion levels. The 100 batches of real-world samples used for evaluation in Section 4.4 are independently held out and never participate in training or fine-tuning. Representative synthetic training samples are illustrated in Figure 8.

#### 4.1.2. Experimental Design & Evaluation Metrics

**Overview of the Experimental Setup.** We use a single NVIDIA RTX 3090 GPU (NVIDIA Corp., Santa Clara, CA, USA) as the training hardware and implement end-to-end training based on PyTorch (v2.1.0). The core structure of the model comprises six components: a ViT encoder (4 Transformer blocks, embedding dimension 144, 8 attention heads), a two-dimensional Haar wavelet decomposition, an FE module, sequential-accumulation multi-view memory fusion, and a sequence recognition head together with a discriminator head. The total number of training epochs is 200, the batch size is 512, and the Adam optimizer is used (learning rate $1\times 10^{-3}$, weight decay $2\times 10^{-5}$) together with a cosine annealing learning-rate schedule ($T_{\max}=200$). The complete set of hyper-parameters is provided in Table 1. Notably, the proposed model contains only approximately 1.9M parameters, which is substantially fewer than mainstream Transformer-based recognizers (e.g., about 62M for TrOCR and 14.05M for the CLIP-based baseline), because the wavelet transform introduces no learnable parameters and its O(N) computational overhead is negligible.

**Loss Function.** The loss function of the model consists of two parts: the sequence recognition loss $L_{\mathrm{CTC}}$ (connectionist temporal classification, CTC) and the discriminator loss $L_{\mathrm{BCE}}^{\mathrm{disc}}$ (binary cross-entropy):

(15) $$L_{\mathrm{total}}=L_{\mathrm{CTC}}\left(P_{\mathrm{char}},S_{\mathrm{true}}\right)+\lambda \cdot L_{\mathrm{BCE}}\left(p_{\mathrm{disc}},y\right),\;\lambda =0.1,$$

where $P_{\mathrm{char}}\in R^{18\times 68}$ is the character probability distribution output by the sequence recognition head, $S_{\mathrm{true}}$ is the ground-truth license plate string, $p_{\mathrm{disc}}$ is the probability of “whether the discriminator can already output the complete license plate,” and y∈{0,1} is the corresponding supervision label.

**Evaluation Protocol.** The evaluation of license plate recognition capability typically requires comprehensively considering multiple dimensions to ensure that the system can recognize license plate information accurately and efficiently under different conditions. This study takes recognition accuracy as the core criterion: this metric reflects the system’s ability to completely and accurately recognize the license plate number, and is computed via the edit distance between the predicted string and the ground-truth string:

(16) $$\mathrm{FMR}=\frac{1}{K}\sum_{k=1}^{K}1\;\left(L(S_{k}^{\mathrm{pred}},S_{k}^{\mathrm{true}})=0\right),$$

where the full-match rate (FMR) measures the overall consistency between the predictions and the ground truth, and a full match requires that the character order and content be completely aligned [49].

#### 4.1.3. Occluded License Plate Recognition Baselines

To comprehensively evaluate our proposed method, we select six representative models from three major categories as baselines: dedicated license plate recognition networks, Transformer-based or industrial-grade general-purpose OCR frameworks, and advanced feature fusion architectures. Specifically, we select LPRNet [50], RPNet [51], and EuLPR [52] as representatives of dedicated license plate recognition methods. To compare against general-purpose scene text recognition paradigms, we introduce the Transformer-based TrOCR [53] and the industrial-grade OCR system PP-OCRv5 [54]. In addition, since our method relies on the integration of multi-view information, we also select MFFN [55], originally proposed for camouflaged object detection, and the multi-modal model CLIP [56] for comparison, in order to evaluate the effectiveness of the feature fusion mechanism. For models that can only recognize a single view, we adopt the MVCP [13] majority-voting fusion strategy (combined with a license plate recognition model to improve the efficiency of low-resolution license plate recognition in real-world scenarios) to ensure the fairness of the experiments.

The baseline models are introduced as follows:

1. **LPRNet [50]**: Proposed by Zherzdev et al., LPRNet is a lightweight, segmentation-free convolutional neural network. It employs a dedicated wide-body architecture to extract contextual features and uses the CTC loss for end-to-end recognition, serving as a standard baseline for real-time license plate recognition tasks.
2. **RPNet [51]**: Proposed by Xu et al., RPNet adopts a progressive approach. It integrates license plate localization and recognition within a unified framework and uses a rectification module to handle geometric distortion, achieving excellent performance in license plate recognition under unconstrained scenarios.
3. **EuLPR [52]**: EuLPR represents an efficient unified license plate recognition framework. It focuses on striking a balance between recognition accuracy and computational efficiency, leveraging deep neural networks to extract robust features from complex backgrounds, and serves as an efficient baseline in this field.
4. **TrOCR [53]**: Developed by Li et al. (Microsoft), TrOCR is a Transformer-based end-to-end optical character recognition model. By leveraging pre-trained image Transformers and text Transformers (e.g., RoBERTa), it treats character recognition as a sequence-to-sequence generation task, providing superior semantic understanding capability.
5. **PP-OCRv5 [54]**: Proposed by Du et al. (PaddlePaddle), PP-OCRv5 is the latest iteration of this industrial-grade OCR system. It integrates multiple strategies such as an ultra-lightweight backbone, data augmentation, and knowledge distillation. This model is chosen to benchmark our method against a highly optimized, state-of-the-art general-purpose OCR engine.
6. **MFFN [55]**: The Multi-view Feature Fusion Network (MFFN) was originally proposed for camouflaged object detection and excels at aggregating information from different views or modalities. We include it as a baseline to rigorously evaluate the model’s feature fusion capability when processing multi-view image sequences, and to verify whether our temporal fusion strategy outperforms generic fusion mechanisms.
7. **CLIP [56]**: A large-scale vision-language pre-training model proposed by OpenAI, which builds general feature representations through image-text contrastive learning. Although it possesses strong zero-shot transfer capability, it is not designed for character-level recognition tasks. In this paper, it is used as a general representation baseline to evaluate the limitations of general-purpose multi-modal models on the fine-grained, structure-sensitive task of occluded license plate recognition, thereby highlighting the necessity of our method in terms of domain adaptation and structural modeling.

### 4.2. Evaluation of Feature Extraction Robustness

First, regarding the choice of wavelet basis type, and inspired by prior research, this paper selects the Haar wavelet as the transformation basis for the discrete wavelet transform. Related studies have shown that the Haar wavelet has the characteristics of a simple structure, low computational overhead, and sensitive responses to local edges and high-frequency details, making it suitable for the multi-scale feature decomposition of noisy, occluded, or structurally fragmented images [42,43,57,58]. At the same time, spatial-domain fusion is adopted as the sub-band fusion strategy. While ensuring high model performance, this combination also possesses good training stability and computational efficiency, providing a solid foundation for the optimization of the entire “ViT + recurrent memory + wavelet transform fusion” framework.

#### 4.2.1. Comparison of Convergence Speed

To analyze the impact of the wavelet-domain transformation on the model optimization process, this paper first focuses on the convergence efficiency of the model under different occlusion conditions. Specifically, we use the training dynamics of FMR as the basis for analysis to examine whether the wavelet-domain representation can help the model focus more quickly on stable discriminative information, thereby improving optimization efficiency and laying the foundation for robust recognition under complex occlusion scenarios.

**Figure 9:**
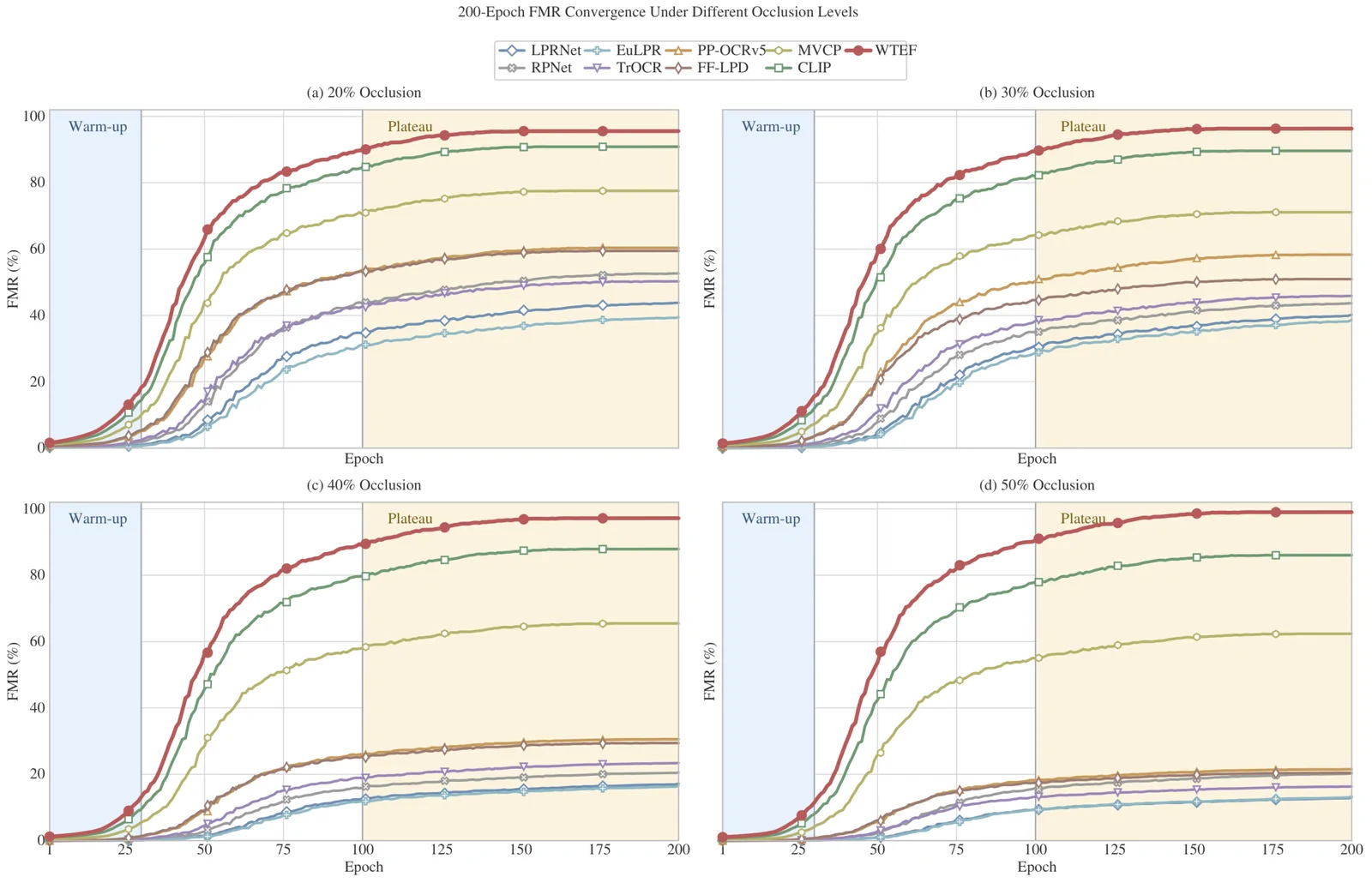
Convergence dynamics of FMR under different occlusion levels (20–50%) over 200 training epochs. Each subplot corresponds to one occlusion level; curves are color-coded per method, consistent with Figure 10.

From the convergence process of the FMR (Figure 9), it can be seen that the proposed method exhibits higher optimization efficiency under occlusion conditions of 20, 30, 40, and 50%. Under the same training budget, WTEF completes the transition from initial representation learning to stable discriminative modeling in fewer epochs, which is specifically reflected in a shorter warm-up phase, a larger slope of performance growth, and an earlier entry into the steady-state plateau region. In contrast, representative baselines such as CLIP, MVCP, and PP-OCRv5 generally require more training rounds to gradually approach their respective optimal states, and their later-stage convergence under high occlusion still exhibits relatively obvious lag. The above phenomena indicate that the wavelet-domain transformation can effectively improve the optimization efficiency of the model and shorten the number of training rounds required to reach stable convergence.

The fundamental reason for this advantage is that the wavelet-domain representation can further decompose spatial features into low-frequency structural information and high-frequency detail information, enabling the originally coupled global license plate contour and local character strokes in multi-view occluded samples to obtain a clearer decoupled representation. Compared with directly performing feature fusion in the spatial domain, this transform-domain modeling approach can more effectively alleviate the feature-aliasing problems caused by occlusion, local misalignment, and noise perturbation, improving the consistency of cross-view features and the stability of gradient propagation. Therefore, the model can obtain a clearer optimization direction more quickly in the early training stage and enter the stable convergence phase earlier. Meanwhile, the low-frequency sub-band in the wavelet domain provides a stable global prior for the overall license plate structure, while the high-frequency sub-bands preserve fine-grained discriminative information such as character edges and stroke textures. The two complement each other during the progressive memory fusion process, enabling the model not only to possess higher training stability but also to continuously recover key recognition cues under occlusion, ultimately providing support for the subsequent improvement in accuracy.

#### 4.2.2. Recognition Accuracy Under Occlusion

To empirically justify the wavelet basis selected in Section 4.2.1, we first compare four candidate bases—db4, bior2.2, sym4, and Haar—within the proposed framework. Table 2 reports the FMR under the same four occlusion levels. The Haar basis consistently achieves the highest FMR, and its advantage widens with occlusion: at 50%, Haar reaches 99.03%, exceeding db4 (97.27%), sym4 (95.96%), and bior2.2 (94.42%). This confirms that the compact support and step-edge sensitivity of Haar best preserve character-level discriminative cues under occlusion, supporting its adoption in the cross-method comparison.

**Figure 10:**
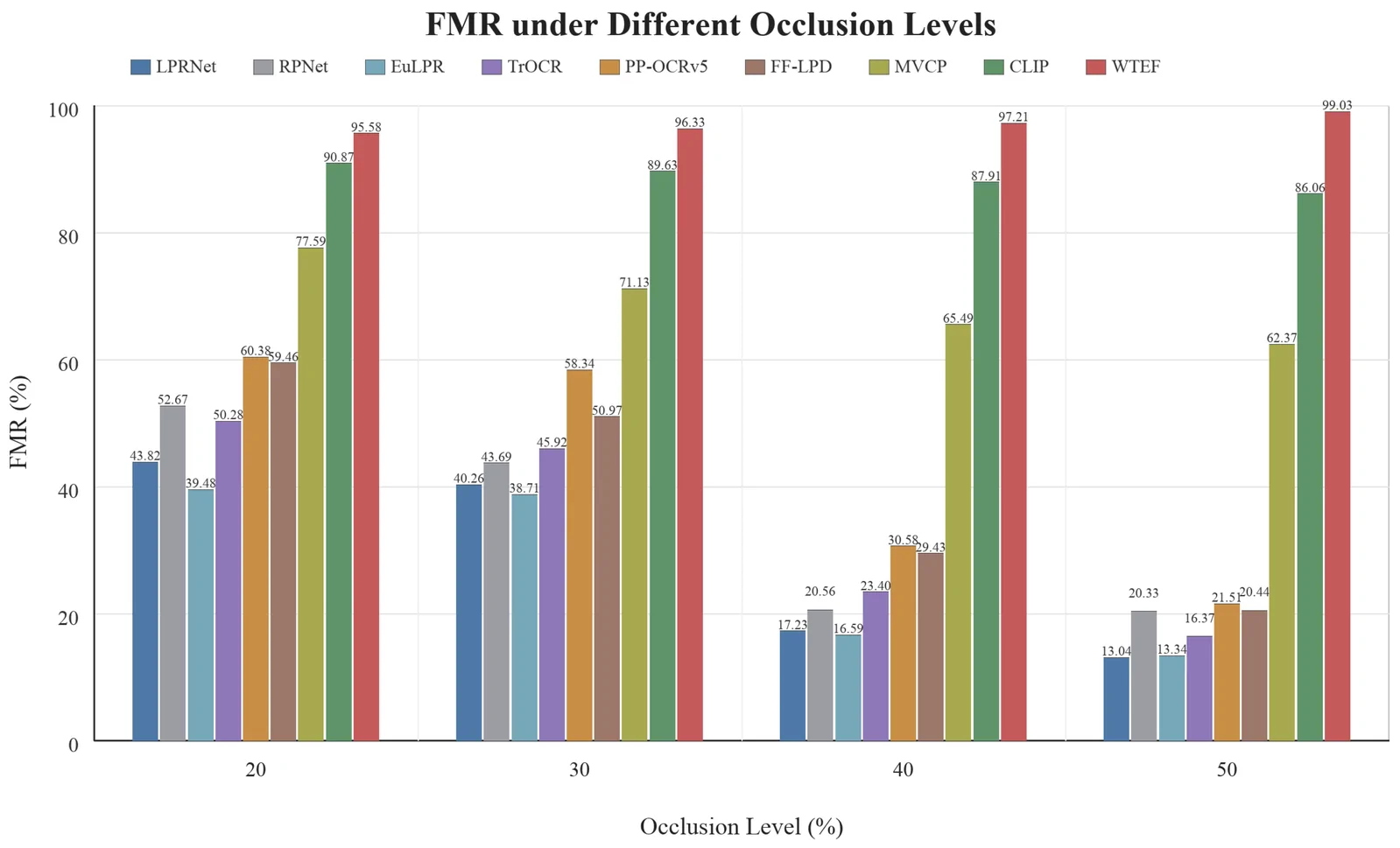
FMR comparison under different occlusion levels (20–50%) at the final training epoch (epoch 200), corresponding to the endpoints of the convergence curves in Figure 9; color coding per method is consistent across both figures.

To comprehensively evaluate the whole-plate recognition performance of the proposed method under different occlusion conditions, this paper uses FMR as a unified evaluation metric across four occlusion levels of 20%, 30%, 40%, and 50%, and systematically compares dedicated license plate recognition models, general-purpose OCR models, and multi-frame fusion models; the quantitative results are shown in Figure 10. Overall, as the occlusion level increases, the FMR of most compared methods drops significantly, indicating that occlusion undermines the structural integrity of the license plate sequence and the consistency of whole-plate recognition. In contrast, the proposed method achieves the best results across all four occlusion levels and consistently maintains extremely high and stable recognition performance. In particular, under the most challenging 50% occlusion condition, WTEF still achieves an FMR of 0.9903, demonstrating excellent anti-occlusion robustness and feature recovery capability.

The experimental results show that traditional dedicated license plate recognition models exhibit particularly pronounced performance degradation under high occlusion. Taking LPRNet as an example, under 50% occlusion, its FMR is only 0.1304, indicating that such models struggle to effectively recover the missing information when character regions are occluded, and tend to misclassify occluded characters as background or produce sequence truncation, thereby leading to whole-plate recognition failure. General-purpose OCR models exhibit a certain degree of competitiveness under mild occlusion, but as the occlusion level deepens, their ability to recover license plate character semantics and sequence structure declines significantly. For example, the FMR of PP-OCRv5 under 50% occlusion is only 0.2151, indicating that relying solely on spatial-domain visual patterns makes it difficult to stably accomplish whole-plate reconstruction under scenarios of severe information loss.

Multi-frame methods are generally superior to single-frame dedicated LPR models and general-purpose OCR baselines, but they still exhibit obvious performance bottlenecks under high-occlusion scenarios. CLIP is the strongest baseline among all compared methods, with FMRs of 0.9087, 0.8963, 0.8791, and 0.8606 under 20, 30, 40, and 50% occlusion, respectively; this indicates that the proposed method can more effectively maintain character structure, recover local semantics, and preserve global sequence consistency under complex occlusion conditions.

In summary, the advantage of the proposed method lies not only in the improvement of whole-plate matching accuracy, but also in its capability for the joint modeling of license plate structural information and sequence relationships. Through wavelet-domain transformation and progressive multi-view memory fusion, the model can stably extract low-frequency structural priors and high-frequency detail cues across different views, thereby achieving more reliable feature reconstruction and whole-plate discrimination when the information in the occluded regions is incomplete. Therefore, under different occlusion levels, the proposed method consistently demonstrates stronger whole-plate recognition capability and better anti-occlusion robustness.

It is worth noting that the FMR of the proposed method increases slightly as the occlusion ratio rises from 20% to 50%. This trend stems from the nature of the task: license plate recognition is essentially a character-wise classification problem, in which the decision relies on the discriminative key strokes of each character rather than its complete appearance. Within the 20–50% range, the core discriminative features of the characters are largely preserved, while occlusion simultaneously masks out background textures, reflections, and stains that would otherwise inject inconsistent high-frequency noise into cross-view fusion. The rising character accuracy (CA) reported in Table 3 directly evidences this mechanism: even when the occluded area is enlarged by 2.5 times, CA still increases from 95.95% to 99.55%. Beyond 50%, however, the physical loss of character-critical strokes dominates and the performance degrades accordingly.

More generally, degradations such as illumination variation, blur, and occlusion are isomorphic forms of information loss: occlusion (y=M⊙x), blur (y=h∗x), illumination modulation (I=L×R) [59], and noise (y=x+n) are all approximately linear information-loss operators, differing only in where the loss occurs—occlusion zeroes out localized full-band regions, blur attenuates the high-frequency sub-bands, and illumination modulates the low-frequency sub-band. In the wavelet domain, these degradations uniformly manifest as missing or attenuated information in specific sub-bands, i.e., sub-band-level occlusion. Unifying heterogeneous degradations as information corruption within a single framework has been validated in the image restoration literature [60,61,62]. Our wavelet-domain progressive fusion compensates for the information loss itself rather than any specific corruption pattern, and owing to the linearity of the DWT (Section 3.3), spatial-domain linear degradations remain isomorphic after the transform, which explains the consistent robustness of the proposed method across both synthetic occlusion and the diverse acquisition conditions present in the data.

#### 4.2.3. Impact of View Count on Recognition

To determine how many occluded views are actually required, we evaluate the average number of views needed for correct recognition under four occlusion levels, as reported in Table 4. The required view count increases monotonically with the occlusion ratio: only 2.2 views on average suffice at 20% occlusion, whereas 4.8 views are consumed at the most challenging 50% level. Notably, across all levels the actual consumption stays below the maximum budget of N=5, which validates the discriminator-guided adaptive stopping mechanism: easy samples are resolved with few views, while heavily occluded samples automatically exploit more observations. Compared with a fixed five-view input, the adaptive scheme reduces view consumption by up to 56.0%, thereby lowering both computational cost and latency without sacrificing recognition accuracy.

#### 4.2.4. Spatial Sensitivity Under Character-Critical Occlusion

Traditional occlusion rates typically use only the proportion of occluded pixel area as the metric, implicitly assuming that different spatial positions in an image contribute equally to the recognition task. As a result, they can only reflect “how much is occluded,” but find it difficult to further characterize “where the occlusion occurs” and “whether the occlusion destroys key discriminative information.” However, for license plate recognition tasks, the character bodies, key strokes, and inter-character structural relationships typically carry stronger semantic information, whereas the peripheral background regions contribute relatively little to whole-plate discrimination. Therefore, under the same occlusion ratio, if the occlusion is concentrated on character-critical regions, the damage it causes to recognition performance is often significantly greater than that of ordinary random occlusion. To more accurately evaluate the model’s sensitivity to damage in key character regions, this paper further constructs a Character-Critical Occlusion (CCO) experiment and uses FMR as a unified evaluation metric for comparative analysis:

(17) $$o_{w}=\frac{\sum_{i,j}M\left(i,j\right)A\left(i,j\right)}{\sum_{i,j}A\left(i,j\right)},$$

where M(i,j) is the occlusion mask, taking the value 1 if pixel (i,j) is occluded and 0 otherwise. A(i,j) is the structural importance weight; a larger value indicates that the discriminative information contained at this position is more critical. For the weight A(i,j), we adopt:

(18) $$A\left(i,j\right)=\left\{\begin{array}{cc} w_{\mathrm{bg}}, & \left(i,j\right)\in \Omega_{\mathrm{bg}}\\ w_{\mathrm{plate}}, & \left(i,j\right)\in \Omega_{\mathrm{plate}} \end{array}\right.\;w_{\mathrm{plate}}>w_{\mathrm{bg}}.$$

In the experiments, we adopt $w_{\mathrm{plate}}=1.0$ and $w_{\mathrm{bg}}=0.2$.

**Figure 11:**
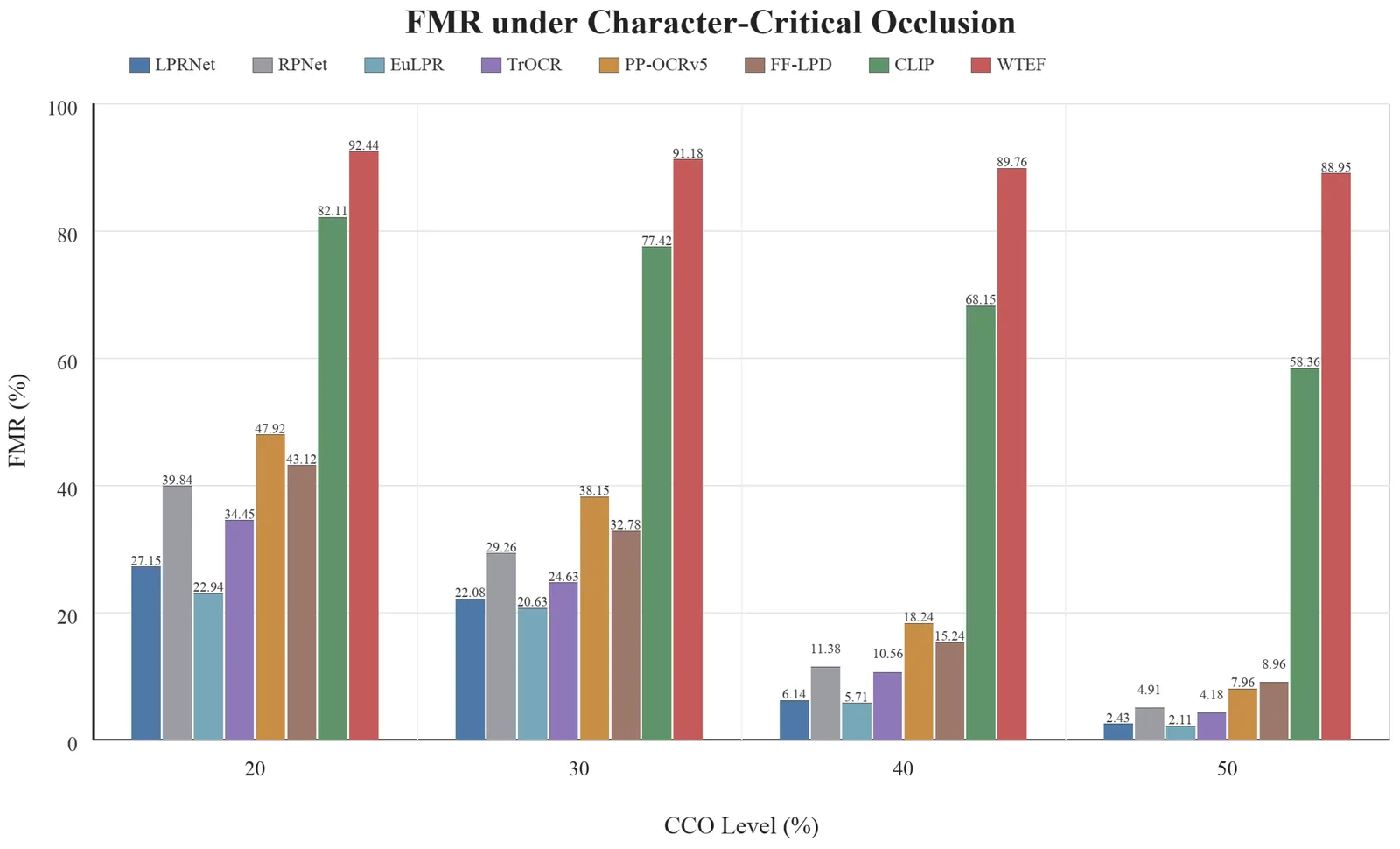
FMR comparison under character-critical occlusion (CCO), where occlusion is preferentially applied to character-body regions rather than random positions. All results are evaluated at epoch 200 with the same trained model as in Figure 10.

Figure 11 presents the FMR results of different methods under 20, 30, 40, and 50% CCO conditions. It can be seen that when occlusion is preferentially applied to character-critical regions, the whole-plate matching performance of all methods declines to varying degrees, indicating that the character body regions carry a higher information load for license plate recognition. In particular, under high-intensity CCO conditions, single-frame dedicated LPR models and general-purpose OCR models degrade markedly. For example, the FMR of LPRNet under 50% CCO is only 2.43%, and that of PP-OCRv5 also drops to 7.96%, indicating that once the key strokes and character structures are occluded, models relying solely on local spatial texture struggle to accomplish reliable sequence recovery.

Multi-frame methods still exhibit certain advantages under CCO conditions, but the differences among models are obvious. As the strongest baseline, CLIP achieves FMRs of 82.11%, 77.42%, 68.15%, and 58.36% under 20%, 30%, 40%, and 50% CCO conditions, respectively, with performance continuously declining as the key-region occlusion increases. In contrast, the proposed method reaches 92.44, 91.18, 89.76, and 88.95% under the same conditions, surpassing CLIP by 10.33, 13.76, 21.61, and 30.59 percentage points, respectively, with the advantage further widening under high-occlusion conditions.

The above results indicate that the degradation of model performance depends not only on the size of the occluded area, but more importantly on whether the occlusion directly destroys the core discriminative information at the character level. The proposed method can still maintain a high FMR under CCO conditions, indicating that wavelet-domain transformation and progressive multi-view memory fusion can more effectively exploit the remaining visible regions and cross-view complementary cues to recover character structure and sequence consistency. Therefore, the proposed method not only possesses strong robustness under ordinary occlusion scenarios, but also demonstrates more stable whole-plate recognition capability in the more challenging scenario where character-critical regions are damaged.

#### 4.2.5. Performance Boundary Under Extreme Occlusion

**Figure 12:**
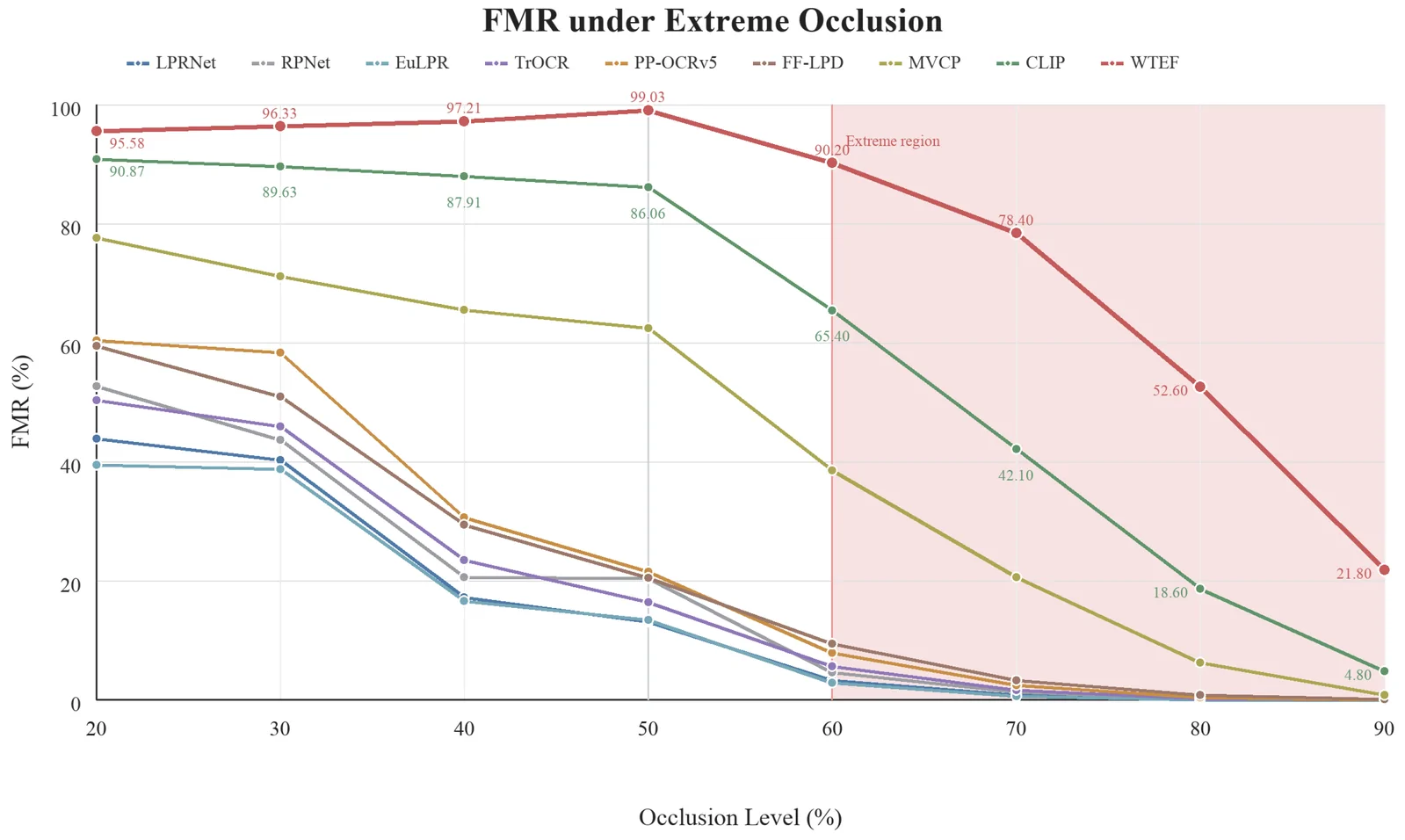
Performance boundary under extreme occlusion (60–90%, shaded region). All results are evaluated with the same trained model (epoch 200) and the identical occlusion protocol; the shaded region denotes the stress-test interval beyond the main evaluation range (20–50%), and 50% marks the approximate performance turning point.

To further investigate the recognition limits of the proposed method beyond the standard experimental range, this paper extends the occlusion analysis from the medium-to-high occlusion interval of 20–50% to the extreme occlusion interval of 60–90%, and uses FMR as a unified evaluation metric. As shown in Figure 12, as the occlusion level continues to increase, the FMR of all methods generally exhibits a downward trend, indicating that as the visible character information continues to decrease, the difficulty of whole-plate-level sequence recovery rises significantly. Within the main experimental interval of 20–50%, the proposed method consistently maintains a high and stable FMR, indicating that the model can fully exploit the remaining visible regions and multi-view complementary information to recover the license plate structure. In contrast, single-frame dedicated LPR models and general-purpose OCR models degrade more rapidly, with an obvious performance collapse already appearing within the 40–50% occlusion interval.

When the occlusion rate further enters the extreme interval of 60–90% (Figure 12), the performance of all methods continues to decline, but the differences in robustness boundaries among different models become more pronounced. Most single-frame methods reach a near-failure state in FMR after 60% occlusion, indicating that relying solely on single-view local visual patterns makes it difficult to support whole-plate recognition under conditions of severe information loss. Although multi-view or strong-representation baselines such as MVCP and CLIP can delay the performance drop to a certain extent, their FMRs still exhibit significant attenuation as the occlusion rate approaches 80% and 90%, indicating that conventional multi-view fusion or general-purpose visual representations still struggle to adequately recover the character sequence structure under extreme occlusion. In contrast, the proposed method still maintains the best performance within the extreme occlusion interval, and its rate of decline is significantly slower than that of the other compared models. In particular, near 80% occlusion, the baseline methods generally exhibit a clear performance inflection point, whereas the proposed method can still maintain relatively stable whole-plate matching capability. This indicates that under extreme occlusion conditions, the system performance bottleneck is no longer determined solely by conventional feature extraction capability, but rather depends more on whether the model can continuously accumulate cross-view complementary evidence and recover character structure and sequence consistency under conditions of high information loss.

Overall, this experiment reveals the practical performance boundaries of different methods under extreme occlusion scenarios. Through wavelet-domain feature decomposition and progressive multi-view memory fusion, the proposed method can more stably preserve low-frequency structural priors and high-frequency detail cues, thereby possessing stronger feature recovery and whole-plate discrimination capability even when the occlusion level increases substantially. This further verifies the robustness advantage of the proposed method under high occlusion and even extreme information-loss scenarios.

#### 4.2.6. Interpretability Analysis of Progressive Feature Accumulation

**Figure 13:**
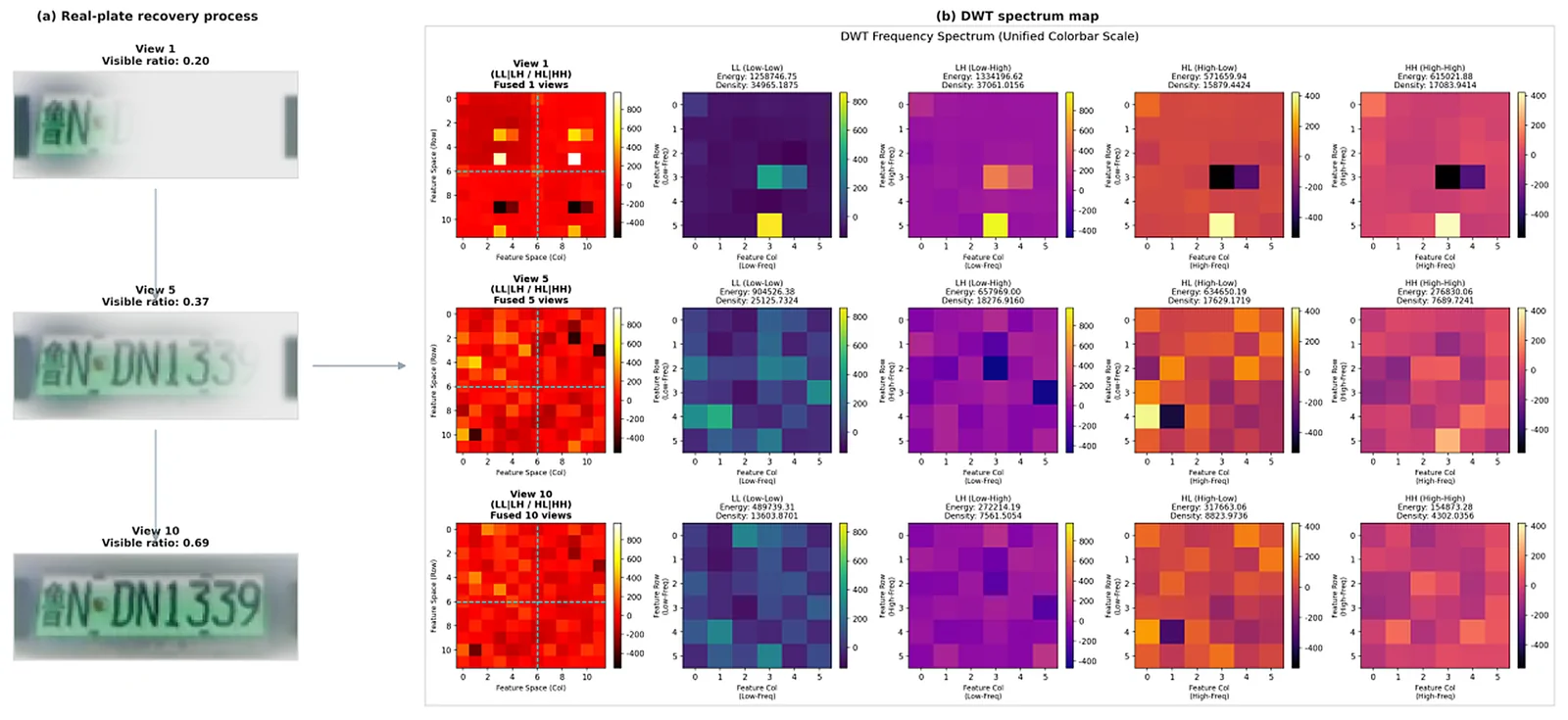
Progressive variation of the fused feature representation and its wavelet spectrum as the number of accumulated views increases from 1 to 10 (each input view remains occluded; panels visualize the IDWT-reconstructed fused features).

We use the consistency of the feature space and the wavelet spectrogram to visualize the process of progressive feature accumulation during recognition. As shown in Figure 13, to further reveal the model’s process of license plate feature extraction and fusion under multi-view inputs, we conduct a visualization analysis of the cumulatively fused DWT spectrogram. As the number of accumulated observation views increases from 1 to 10, the energy distribution, spatial structure, and dynamic range of each sub-band (LL, LH, HL, HH) all exhibit a significant evolutionary trend, reflecting the model’s strong capability in progressively eliminating uncertainty and enhancing feature consistency. The energy of the LL sub-band gradually decreases as the views are fused—from approximately 487,000 at View 1 to approximately 118,000 at View 10. This phenomenon stems from the mathematical property of average fusion (energy $\propto 1/n^{2}$), and also reflects a significant improvement in the signal-to-noise ratio (SNR): the low-frequency components across multiple views tend toward consistency, the noise is suppressed through averaging, while the stable main structure of the license plate is preserved. The LH, HL, and HH sub-bands represent detail information such as edges and textures. As the number of accumulated observation views increases, their energy likewise decreases, but at a faster rate, indicating that high-frequency noise is effectively suppressed while the genuine edge features are preserved owing to multi-view consistency: common knowledge is extracted from redundant observations, achieving a leap from uncertain perception to deterministic representation.

In the early recognition stage, the features are rich in noise and have high energy, but the structure is unstable and the edges are blurred, manifesting as “perceptual chaos.” As the number of input views increases, pattern convergence begins to emerge, the LL sub-band structure takes initial shape, and the edges gradually come into focus, entering the “information integration” stage. When a sufficient amount of view information is input, the features tend to saturate, the spatial patterns lock in, the edges become clear, and the energy converges, completing the “information confirmation.” This process not only verifies the effectiveness of our method in multi-view fusion, but also reveals the intrinsic mechanism by which it maintains semantic consistency in complex scenarios: through cross-view consistency constraints, the model actively filters out incidental perturbations and preserves and strengthens the invariant features of the real physical object.

Furthermore, combined with the CCO experimental results, it can be further demonstrated that the progressive spectral convergence process in Figure 13 not only reflects the noise suppression effect brought about by multi-view fusion in the wavelet domain, but also reveals the cross-view reconstruction mechanism of discriminative information under the condition that character-critical regions are damaged. Compared with ordinary occlusion, CCO leads to a more pronounced loss of low-frequency structure and a more dispersed high-frequency edge response in the early feature representation, thereby increasing the difficulty of feature recovery. However, as the number of accumulated views increases, the proposed method can still progressively recover a stable low-frequency contour and a consistent high-frequency structure, causing the feature representation to converge from an uncertain state to a clear, discriminable state. This is consistent with the result that the proposed method still maintains the smallest performance degradation under CCO conditions, indicating that the proposed progressive fusion mechanism can effectively cope with the collapse of information in key regions.

### 4.3. Ablation Study

**Figure 14:**
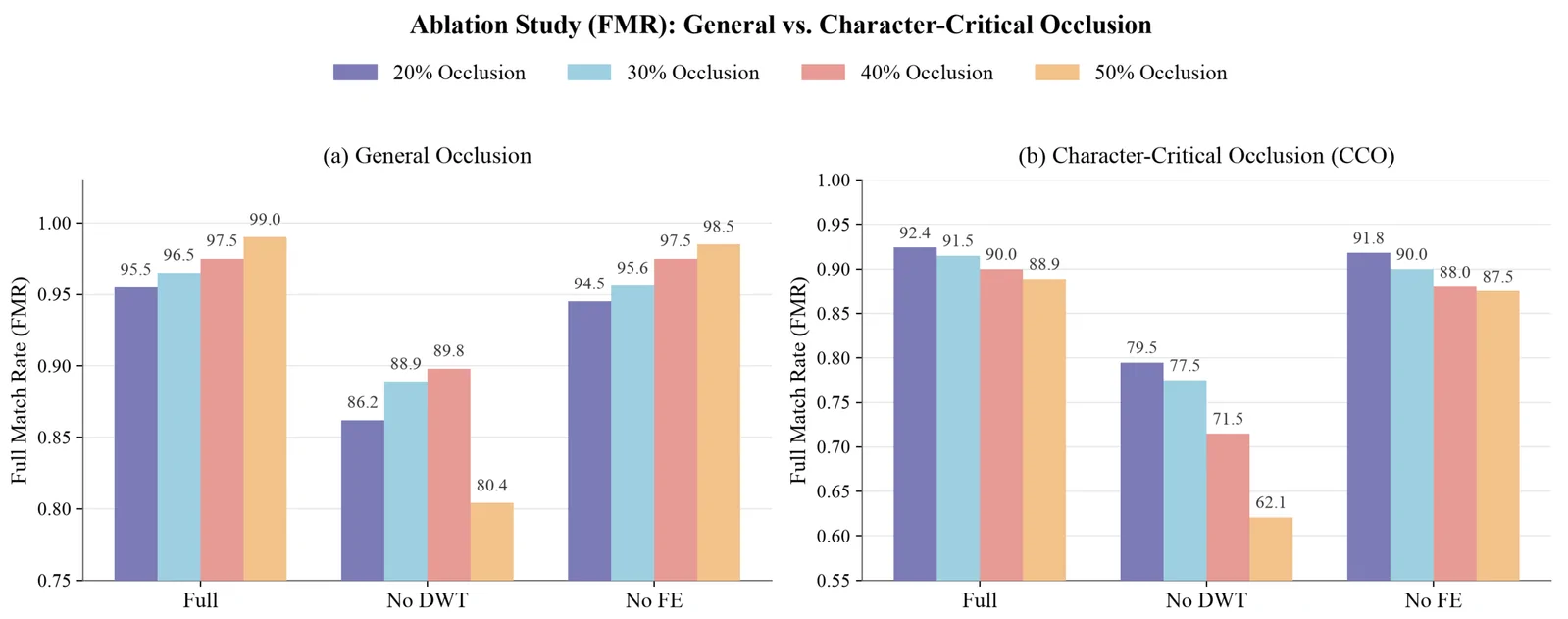
Ablation study: general vs. character-critical occlusion. (**a**) FMR with the FE module removed (No-FE) vs. the full model under general occlusion; (**b**) FMR with the DWT module removed (No-DWT) vs. the full model under character-critical occlusion (CCO). All results are evaluated at epoch 200.

To systematically evaluate the effectiveness of the key modules in the proposed multi-view license plate recognition model, this paper conducts ablation experiments on the discrete wavelet transform and the feature enhancement component, respectively, under four occlusion ratios (20%, 30%, 40%, 50%). The experimental results are shown in Figure 14.

The DWT demonstrates significant and irreplaceable value in high-occlusion scenarios. After removing the DWT, the model performance degrades sharply as the occlusion level intensifies: under mild occlusion (20%), the FMR only drops slightly; when the occlusion increases to 50%, the FMR drops substantially by nearly 19 percentage points (99.03% → 80.42%). This trend indicates that, by mapping spatial-domain features into the frequency domain, the DWT effectively preserves the high-frequency details and structural information of the occluded regions, providing key discriminative cues for multi-view fusion under extreme conditions, and significantly improving the robustness and recovery capability of the model. In contrast, the gain from FE is relatively limited, exhibiting a characteristic of diminishing marginal returns: after removing FE, the FMR loss of the model at all occlusion levels is small; for example, under the most challenging 50% occlusion, the FMR still remains at a high level of 98.48%. This indicates that the currently adopted feature enhancement strategy contributes little directly to the overall matching accuracy; however, it is worth noting that the continuous optimization of FMR by FE indicates that it effectively enhances the consistency and completeness of the sequence-level feature representation. Considering the diverse license plate morphologies in real traffic scenarios, the fine-grained feature modulation capability provided by FE is expected to play a greater role in more complex recognition tasks; therefore, FE should not be regarded as a redundant component, but rather as a robust design component oriented toward future task expansion.

To further analyze the role of the key modules under character-critical occlusion conditions, this paper conducts supplementary ablation experiments on the DWT and FE components under the CCO setting. Consistent with the conclusions under ordinary occlusion conditions, the DWT still plays a significant and irreplaceable role in scenarios where key regions are damaged, and its importance is further enhanced: as the CCO ratio increases from 20% to 50%, removing the DWT leads to a continuous and obvious degradation in the FMR—from 92.44% to 79.54% under 20% CCO, and further to 62.05% under 50% CCO. Compared with the complete model, the performance collapse of No-DWT under high-occlusion key-region conditions is more pronounced, indicating that when the character bodies and their key structures are damaged first, relying solely on spatial-domain features makes it difficult to stably preserve effective discriminative information, whereas the frequency-domain decomposition capability provided by the DWT can more effectively preserve residual structural cues and provide recoverable high-frequency details and contour information for the subsequent multi-view fusion.

Overall, the CCO ablation experiments further indicate that, after the character-critical regions are occluded, the maintenance of the model’s FMR depends more heavily on the joint mechanism of “frequency-domain structure preservation and progressive multi-view fusion.” Among them, the DWT remains the core component supporting the model’s robustness under high-difficulty occlusion conditions, while FE provides an additional feature-stabilization effect on this basis. The FE module is intentionally designed as a lightweight refinement stage rather than the main performance driver; its contribution is expected to be more pronounced under complex real-world degradations. This result indicates that, in the more challenging scenario of character-critical occlusion, the proposed architecture is not only robust to general occlusion, but also able to maintain a strong full-string recovery capability when the key discriminative information is damaged, thereby further verifying the rationality and expansion potential of the proposed method’s design.

In summary, the multi-view, domain-transform, and fusion architecture remains effective under high occlusion. Specifically, the memory fusion in the wavelet domain is the cornerstone of the model’s success; the DWT-domain transformation plays a key role in high-occlusion scenarios, and especially when there are high requirements on FMR under highly complex, high-traffic conditions, it can help the model break through to a higher upper bound of the full-match rate.

### 4.4. Evaluation on Real-World Data

**Figure 15:**
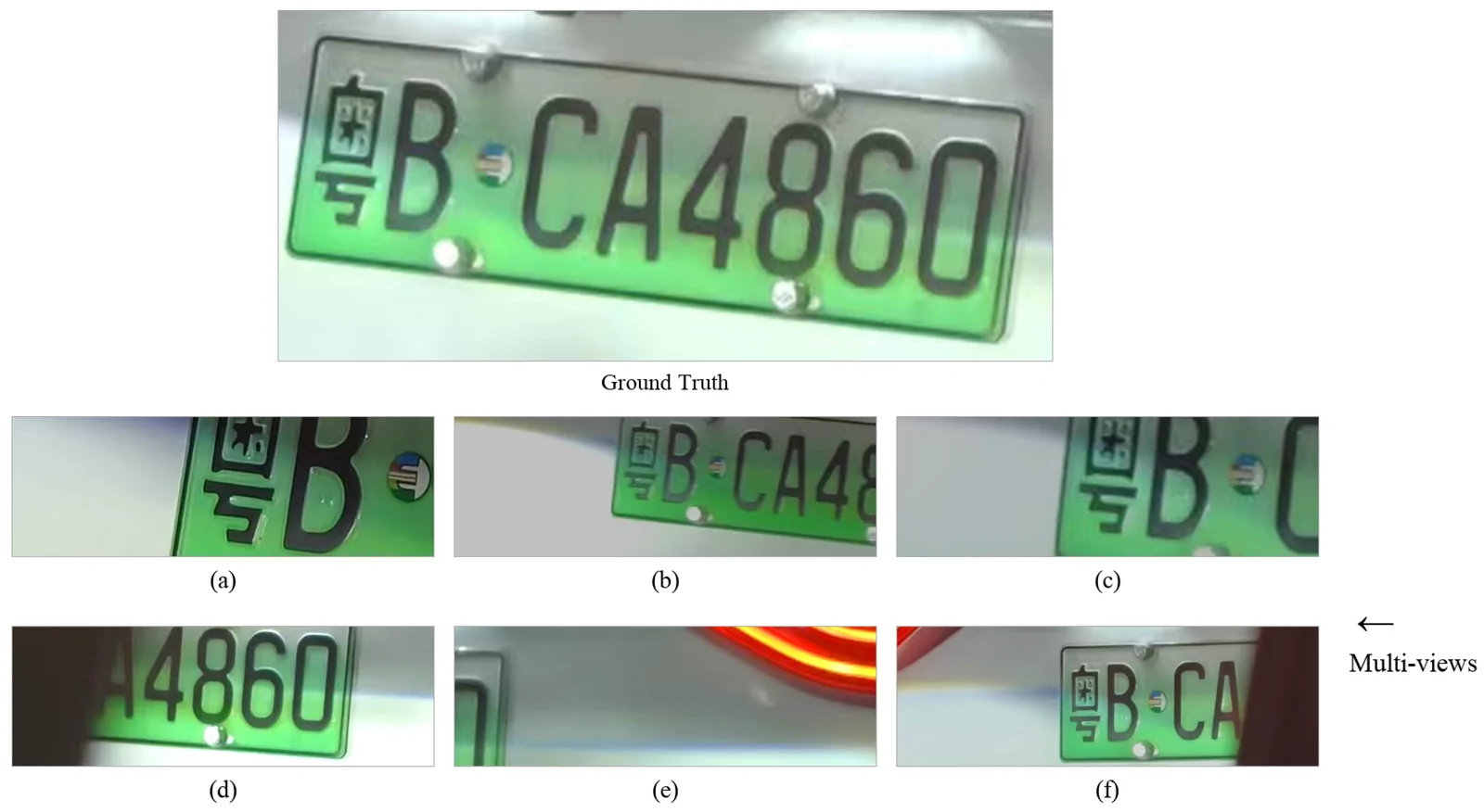
Real-world multi-view occluded license plate samples. The top panel shows the ground-truth plate image; (**a**–**f**) show six views of the same plate captured at an urban traffic intersection, in which different character segments are visible under varying viewing angles and occlusion positions, so that no single view contains the complete plate number. The leading character on the plate is a Chinese province abbreviation, an intrinsic component of the Chinese license plate number.

To verify the robustness and feasibility of our method under real-world traffic flow scenarios, we supplement the main experiments with a set of zero-shot generalization tests targeting real road images. We captured and manually cropped 100 batches of occluded license plate images from real urban traffic intersections as real-world benchmark samples; representative examples are shown in Figure 15.

For evaluation, we still use FMR as the metric, consistent with the main experiments; all compared models (LPRNet, RPNet, EuLPR, TrOCR, PP-OCRv5, MFFN, CLIP) directly load the weights obtained from the training set, and the 100 evaluation batches are independently held out—no model undergoes any retraining or fine-tuning on them, so as to verify the robustness boundary and generalization of each method under real road scenarios.

Three trends can be observed from Table 5. First, the traditional single-frame dedicated license plate models (LPRNet, RPNet, EuLPR) achieve FMRs of only 19–21%, mainly because they overfit to fixed character layouts and clean backgrounds on the synthetic training set, struggle to cope with the illumination, reflections, blur, and irregular occlusion of real-world scenes, and lack cross-view evidence accumulation, so that a single misjudgment causes the entire string to fail. Second, the general-purpose OCR route (TrOCR, PP-OCRv5) achieves FMRs of approximately 39–41%, with stronger character-level generalization, but still suffers from a domain gap with respect to Chinese license plate fonts and character-set distributions, making it difficult to stably output fully correct character results when characters are incomplete. CLIP, which introduces large-scale vision-language priors, further improves to 66%, verifying the effectiveness of strong priors for cross-domain transfer, but its single-frame paradigm still cannot exploit the information complementarity among multiple views. Third, the proposed method achieves the highest FMR of 84%, significantly surpassing CLIP and improving over the strongest traditional dedicated model by more than 60 percentage points. This improvement mainly stems from the wavelet-domain multi-view progressive fusion: the wavelet-coefficient-level representation is more stable against illumination and compression noise, and the cross-view progressive accumulation can complete the missing parts using other views when characters in one view are occluded or contaminated, thereby still stably outputting a complete license plate string consistent with the ground truth under zero training and zero fine-tuning.

In summary, this section demonstrates, under a strict zero-shot protocol, that the proposed method can be directly transferred to the multi-view occluded license plate recognition task at real urban traffic intersections, achieving an FMR of 84% on 100 batches of real-world samples and significantly outperforming all compared methods, thereby verifying its robustness and feasibility in real traffic flow scenarios.

### 4.5. Limitation Analysis

Although the proposed Wavelet-Enhanced Transformer framework demonstrates excellent license plate recognition performance under highly occluded scenarios, there remain several limitations worth discussing.

The training data in this study is dominated by the CBLPRD-330k synthetic data, complemented by real captured occluded plates. Although the synthetic data are generated via GANs and injected with controllable random occlusion, effectively simulating character loss, they lack the complex physical degradation factors of real-world traffic scenes, such as motion blur, dynamic occlusion, and extreme weather. To mitigate this gap, real captured images are included in training (Section 4.1.1), and we further conducted a strict held-out evaluation on 100 batches of real-world occluded plates (Section 4.4); nevertheless, systematic evaluation under harsher real-world degradations is still required and is left for future work.

The core assumption of this method is that the same license plate appears multiple times in the temporal sequence with different viewpoints/occlusion states, thereby providing complementary information. However, in actual traffic flow, if a vehicle passes at high speed or the camera captures insufficient information, the number of effective observation frames may be inadequate, preventing the recurrent memory module from accumulating sufficient features to complete the reconstruction. For extreme cases such as a single frame being fully occluded or the target disappearing for an extended period, the model lacks an effective robustness mechanism.

## 5. Conclusions and Future Work

To address the difficulty of license plate recognition caused by occlusion arising from complex traffic flow in intelligent transportation systems, this paper, starting from a “progressive recognition” framework, proposes the Wavelet-Enhanced Transformer mechanism. This scheme innovatively shifts the basis of feature fusion from the spatial domain to the wavelet domain, resolving the model’s dependence on positional encoding in the spatial domain and improving the model’s robustness to large-area occlusion.

Specifically, the main contributions of this study are reflected in three aspects. First, we design a novel domain-transform feature fusion mechanism that leverages the time-frequency localization characteristics of the discrete wavelet transform to decouple the fragmentary license plate features under multiple views into semantic features of different frequency bands. This strategy effectively avoids the spatial-position misalignment problem caused by viewpoint variation and occlusion, achieving precise alignment at the semantic level rather than the pixel level. Second, we construct an end-to-end progressive occluded license plate recognition framework that, through a recurrent memory fusion module, dynamically accumulates and integrates the non-occluded-region features from different time steps, thereby still being able to reconstruct the complete license plate sequence when single-frame information is severely missing. Finally, extensive experiments verify the superiority of the proposed method. Under extreme occlusion conditions where the character-missing area is as high as 20% to 50%, our model significantly outperforms existing mainstream methods on the three key metrics of character accuracy (CA), character length accuracy (LA), and FMR, fully demonstrating its strong robustness and high efficiency in complex traffic scenarios.

Although we have achieved breakthrough results, this study still has directions worth further exploration. Future work mainly focuses on the following aspects:

**Improving generalization to real-world scenarios:** The current model is mainly trained and validated on the synthetic dataset (CBLPRD-330k). In the future, we will devote efforts to constructing a large-scale real-world occluded license plate dataset that includes complex real-world degradation factors (such as motion blur, extreme illumination, rainy and foggy weather, and dynamic occluders), and perform model fine-tuning and evaluation on this basis to enhance its generalization performance in practical roadside deployment.

**Edge-computing-friendly model design:** Although this model performs excellently in terms of accuracy, the combination of its ViT backbone and wavelet transform module imposes certain requirements on computational resources. To better adapt to resource-constrained edge devices (such as roadside units (RSUs) or in-vehicle terminals), we will study model lightweighting techniques, including knowledge distillation, network pruning, and quantization, aiming to develop a lightweight WTEF variant that is high-accuracy, low-latency, and low-power, so as to support large-scale real-time deployment.

**Exploring cross-task transfer capability:** This framework is tailored specifically for the license plate recognition task. In the future, we will verify whether its core idea—“domain transformation + progressive fusion”—can be transferred to other intelligent transportation perception tasks, such as fine-grained vehicle-type recognition, vehicle re-identification, or traffic-participant behavior prediction, with the aim of building a general-purpose visual perception foundation model oriented toward occlusion scenarios.

In summary, the Wavelet-Enhanced Transformer proposed in this paper provides an effective pathway for solving the highly challenging problem of occluded license plate recognition in ITS. We believe that, through continuous optimization and extension, this framework has the potential to become a key component of future smart-city traffic visual perception systems.

## Figures and Tables

**Table 1:** Implementation details and hyper-parameters.

Component	Setting
Backbone (ViT Encoder)	4 Transformer blocks, 8 attention heads, embedding dim 144, MLP ratio 4
Patch size	8×8 (Conv2d-based PatchEmbed)
Input resolution	48×128 (H×W)
DWT	2D Haar wavelet, 1-level decomposition
Subband fusion strategy	Spatial concatenation
Sequence head	2-layer MLP: Linear(144 → 288) + ReLU + Dropout(0.1) + Linear(288 → 18 × 68)
Discriminator head	2-layer MLP: Linear(144 → 256) + ReLU + Linear(256 → 1) + Sigmoid
Memory fusion	$M_{t}=\left(X_{t}+\sum_{i=1}^{t-1}M_{i}\right)/t$
Multi-view	N=5
Stop criterion	$p_{t}\geq 0.5$ (Disc), max iteration $T_{\max}=N=5$
Optimizer	Adam $\beta_{1}=0.9,\beta_{2}=0.999$
Initial learning rate	$1\times 10^{-3}$
LR schedule	Cosine Annealing, $T_{\max}=200$ epochs
Weight decay	$2\times 10^{-5}$
Batch size	512 (train & val)
Epochs	200
Loss function	$L=L_{\mathrm{CTC}}+0.1\cdot L_{\mathrm{BCE}}\left(\mathrm{disc}\right)$
CTC blank index	|C|−1=67
Gradient clipping	max-norm 1.0
Image normalization	(pixel − 127.5)/128
Max plate length	18
Hardware	NVIDIA RTX 3090
Random seed	42

**Table 2:** FMR (%) of different wavelet bases under varying occlusion levels.

Occlusion	db4	bior2.2	sym4	WTEF (Haar)
20%	94.62	91.48	93.05	**95.58**
30%	95.18	92.15	93.71	**96.33**
40%	95.84	92.93	94.62	**97.21**
50%	97.27	94.42	95.96	**99.03**

Bold values indicate the best performance at each occlusion level.

**Table 3:** Character accuracy (CA) and FMR (%) of the proposed method under different occlusion levels (epoch 200).

Metric	20%	30%	40%	50%
CA (%)	95.95	96.84	97.42	99.55
FMR (%)	95.58	96.33	97.21	99.03

**Table 4:** Average number of views required for correct recognition under different occlusion levels. The maximum view budget is N=5; the discriminator-guided stopping mechanism adaptively terminates early when sufficient evidence has been fused.

Occlusion Level	20%	30%	40%	50%
Avg. views needed	2.2	2.8	3.5	4.8
View reduction vs. fixed N=5	56.0%	44.0%	30.0%	4.0%

**Table 5:** Zero-shot FMR (%) on real-world multi-view occluded license plates (K=100).

Method	LPRNet	RPNet	EuLPR	TrOCR	PP-OCRv5	MFFN	CLIP	WTEF
FMR (%)	21	19	20	39	41	53	66	**84**

Bold value indicates the best performance.

## Data Availability

The CBLPRD-330k dataset used in this study is publicly available at https://github.com/SunlifeV/CBLPRD-330k (accessed on 24 August 2026). The source code of the proposed WTEF framework is publicly available at https://github.com/Pernod06/Believe-attention (accessed on 24 August 2026).
